# The bone marrow microenvironment of pre-B acute lymphoblastic leukemia at single-cell resolution

**DOI:** 10.1038/s41598-020-76157-4

**Published:** 2020-11-05

**Authors:** Denise Anderson, Patrycja Skut, Anastasia M. Hughes, Emanuela Ferrari, Jennifer Tickner, Jiake Xu, Benjamin H. Mullin, Dave Tang, Sébastien Malinge, Ursula R. Kees, Rishi S. Kotecha, Timo Lassmann, Laurence C. Cheung

**Affiliations:** 1 Telethon Kids Institute, The University of Western Australia, Perth, WA Australia; 2 School of Pharmacy and Biomedical Sciences, Curtin University, Perth, WA Australia; 3 School of Biomedical Sciences, The University of Western Australia, Perth, WA Australia; 4 Department of Endocrinology and Diabetes, Sir Charles Gairdner Hospital, Perth, WA Australia; 5 Division of Genomic Technologies, RIKEN Center for Life Science Technologies, Yokohama, Japan; 6 Department of Haematology and Oncology, Perth Children’s Hospital, Perth, WA Australia

**Keywords:** Acute lymphocytic leukaemia, Cancer microenvironment

## Abstract

The bone marrow microenvironment (BMM) plays a key role in leukemia progression, but its molecular complexity in pre-B cell acute lymphoblastic leukemia (B-ALL), the most common cancer in children, remains poorly understood. To gain further insight, we used single-cell RNA sequencing to characterize the kinetics of the murine BMM during B-ALL progression. Normal pro- and pre-B cells were found to be the most affected at the earliest stages of disease and this was associated with changes in expression of genes regulated by the AP1-transcription factor complex and regulatory factors NELFE, MYC and BCL11A. Granulocyte–macrophage progenitors show reduced expression of the tumor suppressor long non-coding RNA *Neat1* and disruptions in the rate of transcription. Intercellular communication networks revealed monocyte-dendritic precursors to be consistently active during B-ALL progression, with enriched processes including cytokine-mediated signaling pathway, neutrophil-mediated immunity and regulation of cell migration and proliferation. In addition, we confirmed that the hematopoietic stem and progenitor cell compartment was perturbed during leukemogenesis. These findings extend our understanding of the complexity of changes and molecular interactions among the normal cells of the BMM during B-ALL progression.

## Introduction

The microenvironment of cancer is known to play a critical role in cancer progression and contributes to treatment failure or success^1^. In the bone marrow, the microenvironment contains an array of cell types including immune cells, osteoblasts, osteoclasts, endothelial cells, mesenchymal stromal cells and adipocytes which play a key role in regulating hematopoiesis in a non-cell autonomous manner. During leukemia development, tumor cells alter the bone marrow microenvironment (BMM) to favor disease development at the expense of normal hematopoiesis. In acute myeloid leukemia (AML), tumor cells have been shown to reduce osteoblast numbers, with ablation of osteoblasts altering lineage determination of hematopoietic stem cells and promoting leukemia progression in vivo^2^. Furthermore, immune cells in the bone marrow contribute to leukemogenesis. Macrophages and *CSF1R*-expressing myeloid cells have been shown to promote the survival of chronic lymphocytic leukemia cells in vivo and promote AML cell growth, respectively^3,4^.

Rapid progress in the development of single-cell RNA sequencing technologies has permitted the study of cell-specific gene expression in a complex environment at single-cell resolution. It has been used to identify distinct molecular signatures of leukemia stem cells in chronic myeloid leukemia and to determine clonal heterogeneity in T-cell acute lymphoblastic leukemia^5,6^. Recently, single-cell analyses have provided novel insights into the non-hematopoietic stroma, in both non-leukemic murine bone marrow under steady state and stress conditions, and in a MLL-AF9-driven AML model^7,8^. While our understanding of the BMM in myeloid malignancies has advanced markedly over the past decade, little is known regarding the role of the BMM in pre-B cell acute lymphoblastic leukemia (B-ALL), the most common cancer in children. To further investigate, we used single-cell RNA sequencing to characterize the kinetics of the murine BMM during B-ALL progression. We examined the molecular changes of immune cell populations and our results shed light on the complexity of intracellular communication networks and the alteration of hematopoietic lineage in the BMM during B-ALL progression.

## Results

To explore the dynamic changes of the BMM during leukemia progression, we profiled hematopoietic cells from an immunocompetent BCR-ABL1^+^ B-ALL model^9^ by droplet-based single-cell RNA sequencing and examined changes in gene expression and intercellular communication over time. In this model, 1000 BCR-ABL1^+^ cells were transplanted into each non-irradiated recipient, thus avoiding irradiation-induced changes to the microenvironment. Leukemia cells homed to bone marrow and spleen, and mice developed BCR-ABL1^+^ leukemia within 3 weeks. Leukemia cells remained at a low level of < 1% for 10 days, followed by rapid expansion in bone marrow and spleen, and slightly later in blood^9^.

### Cell types in the bone marrow microenvironment

**Figure 1:**
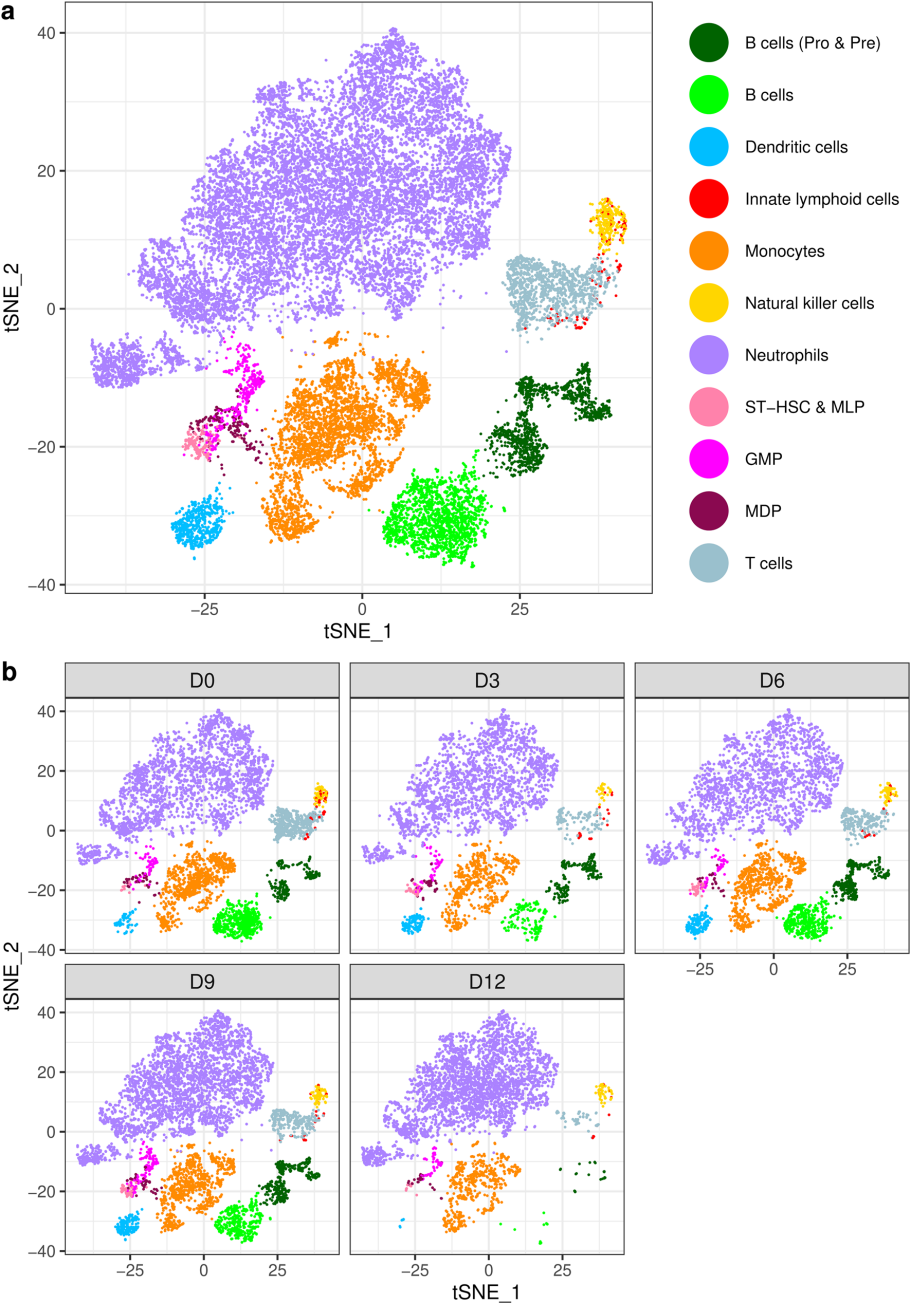
Inferred cell type clusters used in downstream analyses. t-SNE dimensionality reduction plots show all samples (**a**) and individual samples at each time point (**b**). Cells were clustered using Seurat and annotated by *SingleR*. ST-HSC & MLP = short term repopulating cells and multilineage progenitors, GMP = granulocyte–macrophage progenitors, MDP = monocyte-dendritic precursors.

Clustering of the samples with Seurat (Supplementary Fig. 1), followed by cell type classification with *SingleR* revealed 17 distinct cell type clusters (Supplementary Figs. 2 and 3). Depletion of most cell types occurred by day 12 of disease progression (Supplementary Fig. 3, Supplementary Table 1). Seurat cluster 7 (Supplementary Fig. 1) contained a mix of cell types, and cells in this cluster had a much lower number of expressed genes per cell compared to other clusters, so this cluster was excluded from further downstream analysis. We combined pro- and pre-B cells as these cell types substantially overlapped based on their expression profile (Supplementary Figs. 2, 3 and 4). The hematopoietic stem and progenitor cells (HSPCs) identified with *SingleR* are designated as five stem cell populations, the short term repopulating cells (STSL), multilineage progenitors (MLP), granulocyte–macrophage progenitors (GMP), monocyte-dendritic precursors (MDP) and common dendritic precursors (CDP) (Supplementary Table 1). We combined STSL and MLP and thereafter refer to them as ST-HSC & MLP (Supplementary Table 2). We excluded early T cell precursors (ETP) from further analysis due to small numbers (Supplementary Table 1). Many of the cells annotated as common lymphoid progenitor (CLP) B cells and macrophages were in cluster 7 and after exclusion of this cluster there were too few cells from these cell types for analysis. Finally, we excluded cells with discordant annotation (e.g. cells annotated as monocytes that were present in the neutrophil cluster; Supplementary Fig. 2) and this left too few CDP for further analysis. The final cell types and numbers for downstream analysis are shown in Fig. 1 and Supplementary Table 2.

To check the validity of the inferred cell types, we identified conserved marker genes for each cluster (Supplementary Fig. 4). The genes that characterized the B cell cluster included those involved in activation of the immune response (*Iglc2*), and antigen processing and peptide presentation (*H2-Aa*, *H2-Eb1*) in B lymphocytes. Pro-B, pre-B and B cells expressed *Cd79a* and *Ebf1*, which are required for B cell differentiation, proliferation and signaling. Pro- and pre-B cells were marked by expression of *Mzb1* and *Vpreb3*, which are involved in regulation of B cell proliferation and maturation. Dendritic cells were characterized by expression of *Tcf4* which controls dendritic cell lineage specification^10^, *Ccr9* which is involved in dendritic cell maturation^11^ and *Siglech* which is involved in mediation of the immune response^12^. Marker genes for monocytes included *Fn1* and *Ctss* which are involved in monocyte differentiation^13,14^. Natural killer cells were characterized by expression of well-known marker genes *Klrd1*, *Klrk1*, *Klre1* and *Gzma*^15^. Marker genes for HSPCs included *Prtn3* and *Lmo2* which regulate hematopoietic stem and progenitor cell proliferation^16^, *Cdk6* which regulates hematopoietic and leukemic stem cell activation^17^, *Cd34* which may be required for attachment to the bone marrow extracellular matrix and *Ms4a3* which is involved in regulation of the cell cycle and known to be expressed in developing hematopoietic cells. T cells expressed known marker genes *Cd3d* and *Trbc2*. We conclude that in silico annotation of cell types with *SingleR* is accurate, as evidenced by expression of known marker genes, and these cell types will be used for subsequent downstream analysis.

### Differential expression analysis by cell type during disease progression

**Figure 2:**
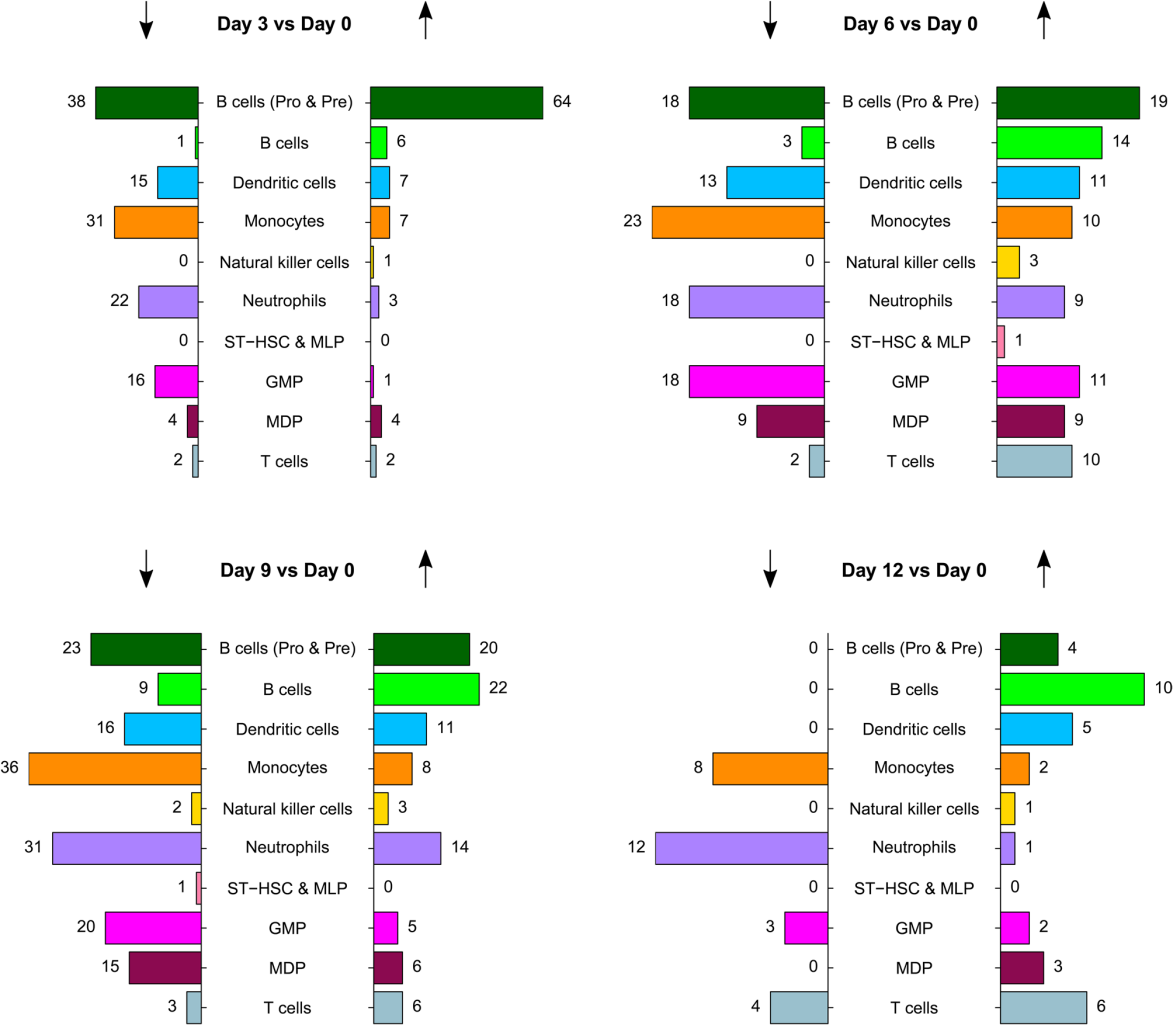
Number of differentially expressed genes by cell type and direction of change (↓ = underexpressed and ↑ = overexpressed) during disease progression. Results are adjusted for multiple testing (Bonferroni correction) and genes have an absolute log fold change of at least 0.4. ST-HSC & MLP = short term repopulating cells and multilineage progenitors, GMP = granulocyte–macrophage progenitors, MDP = monocyte-dendritic precursors.

We compared expression of genes during disease progression to expression at day 0 (Fig. 2, Supplementary Tables 3–6). Dendritic cells, monocytes, neutrophils and GMP had more underexpressed than overexpressed genes at days 3, 6 and 9 compared to day 0. This finding was validated by qRT-PCR of select genes in the neutrophil population, the most abundant white blood cell type in the bone marrow (Supplementary Fig. 5). Our results suggest a reduction in gene expression in response to disease progression.

In contrast to the aforementioned cell types, the largest change in the number of differentially expressed genes was for pro- and pre-B cells at day 3, where increased expression for 64 genes and decreased expression for 38 genes was seen. This included reduced expression of a number of genes that are members of the activating protein 1 (AP-1) transcription factor complex (*Jund*: logFC = − 1.38, *p* = 3.2 × 10^–34^; *Fos*: logFC = − 1.42, *p* = 3.5 × 10^–19^; *Fosb*: logFC = − 1.26, *p* = 7.7 × 10^–13^; *Jun*: logFC = − 0.85, *p* = 2.8 × 10^–10^; *Junb*: logFC = − 0.87, *p* = 0.006). The reduced expression across these genes was still evident when comparing both days 6 and 9 to day 0 for pro- and pre-B cells, although the magnitude of reduction (logFC) was smaller. We also saw a significant reduction in expression for most or all of these genes in dendritic cells, monocytes, neutrophils, GMP and MDP up to day 9 of disease progression, however pro- and pre-B cells were the only cell type showing consistent decreased expression of all five genes over time. At day 12, many of the cell types were depleted due to advanced disease (Supplementary Table 2) and consequently we only saw significantly reduced expression of *Jun* and *Junb* in neutrophils.

Two other genes that were differentially expressed in pro- and pre-B cells at day 3 are *Cdkn2a* (logFC = 0.68, *p* = 1.9 × 10^–25^) and *Tmem119* (logFC = 0.59, *p* = 1.5 × 10^–22^). Both genes were not differentially expressed at the other time points, nor were they differentially expressed in any other cell type. *Cdkn2a* acts as a tumor suppressor in ALL through regulation of the cell cycle^18,19^ and *Tmem119* is involved in osteoblast differentiation^20^. Finally, a number of the differentially expressed genes for pro- and pre-B cells at day 3 were enriched for regulation by RNA binding protein NELFE and transcription factors MYC (Enrichr ENCODE and ChEA Consensus TFs from ChIP-X: NELFE *p* = 6.0 × 10^–12^; MYC *p* = 5.4 × 10^–11^) and BCL11A (Enrichr TF Perturbations Followed by Expression: *p* = 1.2 × 10^–27^). A large study of hepatocellular carcinoma^21^ found that disease progression was associated with oncogenic activation of NELFE which led to enhancement of MYC signaling and global transcriptomic imbalance. Deregulation of BCL11A has been associated with hematopoietic malignancies and AML^22,23^.

For GMP we found reduced expression of long non-coding RNA *Neat1* at all time points except day 12 (D3vsD0: logFC = − 0.73, *p* = 2.1 × 10^–7^; D6vsD0: logFC = − 0.66, *p* = 1.3 × 10^–5^; D9vsD0: logFC  = − 0.71, *p* = 2.4 × 10^–10^). *Neat1* is highly expressed in hematopoietic stem and progenitor cells^24^ and has previously been described as a tumor suppressor in hematological malignancies^25,26^. In summary, we found that most cell types had reduced gene expression during disease progression, contrasting with pro- and pre-B cells which showed the strongest initial response to disease, with more overexpressed than underexpressed genes.

### Intercellular communication networks of the BMM

**Figure 3:**
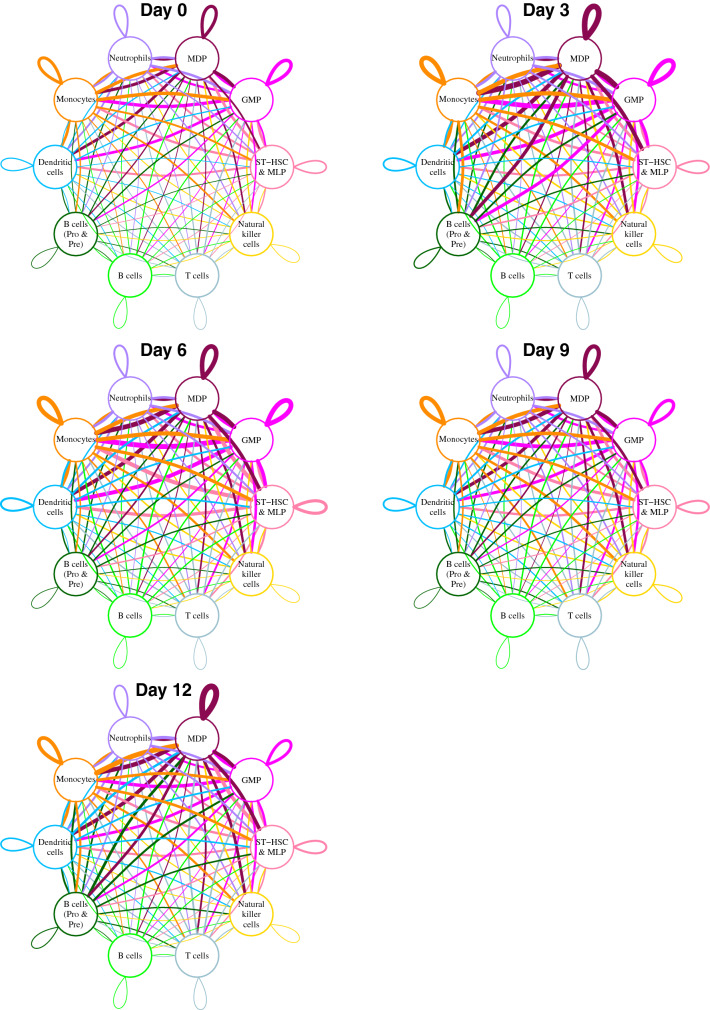
Intercellular communication networks of known ligand/receptor pairs. Edge widths correspond to the number of links between cell pairs or within the same cell type. A link is one-way directional and is defined when a cell type expresses the ligand and another cell type (or the same cell type) expresses the corresponding receptor. ST-HSC & MLP = short term repopulating cells and multilineage progenitors, GMP = granulocyte–macrophage progenitors, MDP = monocyte-dendritic precursors.

To understand how different cell types interact during disease progression we constructed intercellular communication networks of known ligand/receptor pairs (Fig. 3). The edge widths on these networks allow us to visualize the strength of communication of each cell type and how this communication changes during disease progression. The most communicative cells on these networks that also showed the largest changes in communication during disease progression were monocytes and HSPCs. Monocytes showed increased communication with ST-HSC & MLP and MDP at all days compared to day 0 (Supplementary Fig. 6). Ligands expressed by monocytes were primarily enriched for GO Biological Processes (GO-BP) associated with cell migration and positive regulation of phosphorylation at days 6 and 9 (Supplementary Fig. 7). When considering receptors expressed by monocytes, we saw enrichment for GO-BP associated with inflammatory response at day 3 and reduced enrichment for genes involved in extracellular matrix organization, neutrophil-mediated immunity and cellular response to cytokine stimulus at all days compared to day 0 (Supplementary Fig. 8).

For ST-HSC & MLP, increased communication with other cell types was most evident at day 6 (Supplementary Fig. 9). Enriched GO-BP for expressed ligands at day 6 was primarily for cytokine-mediated signaling pathways, though we also saw enrichment for positive regulation of cell migration and phosphorylation (Supplementary Fig. 10). Expressed receptors showed enrichment for GO-BP associated with regulation of apoptotic process and extracellular matrix organization at day 3 and reduced enrichment for genes involved in positive regulation of protein phosphorylation at all days compared to day 0 (Supplementary Fig. 11).

Increases in communication for GMP was primarily seen at days 3 and 6 (Supplementary Fig. 12). Similarly to ST-HSC & MLP, the primary enrichment for expressed ligands was for cytokine-mediated signaling pathways at day 6, and to a lesser extent we saw enrichment for positive regulation of cell migration, positive regulation of phosphorylation and platelet degranulation (Supplementary Fig. 13). When considering expressed receptors, increased enrichment for genes involved in extracellular matrix organization was seen at day 6 (Supplementary Fig. 14). There was also a marked difference when comparing enrichment of GO-BP at day 12 versus all other days. At day 12 there was decreased enrichment of a number of GO-BP, including amongst others, regulation of apoptotic process, leukocyte cell–cell adhesion, cell–matrix adhesion and regulation of B cell proliferation. Conversely there was increased enrichment of GO-BP associated with neutrophil-mediated immunity.

Finally, for MDP, communication increases were seen between most cell types at all days compared to day 0 (Supplementary Fig. 15). For the expressed ligands increased enrichment for many GO-BP was evident, particularly for days 3 and 12 compared to day 0 (Supplementary Fig. 16). These included processes such as cytokine-mediated signaling pathway, regulation of cell migration, positive regulation of cell proliferation, positive regulation of phosphorylation and neutrophil-mediated immunity. For the expressed receptors, the trend was also for increased enrichment of GO-BP at later time points in comparison to day 0 and these included amongst others, positive regulation of protein phosphorylation, neutrophil-mediated immunity, leukocyte cell–cell adhesion, integrin-mediated signaling pathway and receptor-mediated endocytosis (Supplementary Fig. 17).

### RNA velocity analysis

**Figure 4:**
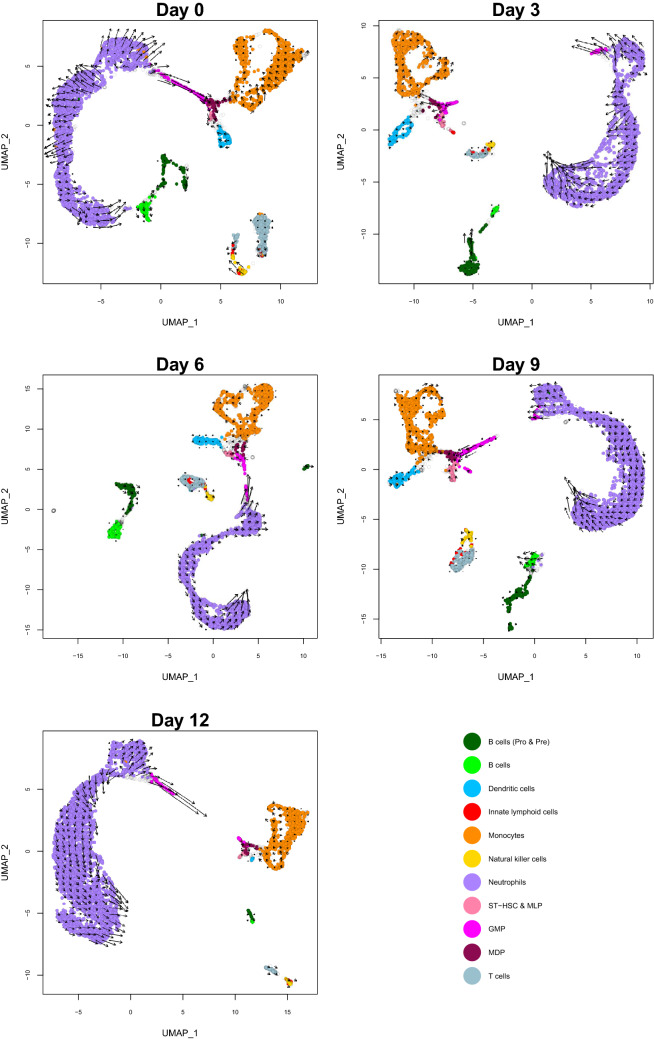
RNA velocity estimates for each cell type over time. Estimates (arrows) are plotted on a Uniform Manifold Approximation and Projection (UMAP) dimensionality reduction. The length of the arrows represents the transcription rate (velocity) and the direction of the arrows points to the inferred future state of the cell based on other cells present on the UMAP plot. Transparent points are those cells that were filtered out as described in “Results” (cell types in the bone marrow microenvironment). ST-HSC & MLP = short term repopulating cells and multilineage progenitors, GMP = granulocyte–macrophage progenitors, MDP = monocyte-dendritic precursors.

To better understand global transcriptional dynamics during disease progression we estimated RNA velocity (transcription rate) for each cell, which also allows inference of the future state of the cell (Fig. 4). For pro- and pre-B cells the strongest velocity was observed for a sub-population of cells at day 3 and this also aligned with our differential expression results where pro- and pre-B cells showed the most differentially expressed genes at day 3. From day 9 onwards velocity is markedly reduced, suggesting an initial response to the disease that is not sustained.

At day 0, GMP had high velocity and their inferred future state showed a continuous direction of movement (trajectory) from neutrophils through to MDP, both of which differentiate from GMP. This trajectory split from day 3 onwards into GMP that are clustered near neutrophils, and those that clustered near ST-HSC & MLP and MDP. Those that clustered near neutrophils had higher velocity than those that clustered near ST-HSC & MLP and MDP. Hence, we found evidence for disruption in global transcriptional dynamics commencing at day 3 of disease progression, particularly for GMP, due to individual cells exhibiting changes in velocity that affect their future state.

### Enumeration of the bone marrow HSPC compartment during leukemia development

**Figure 5:**
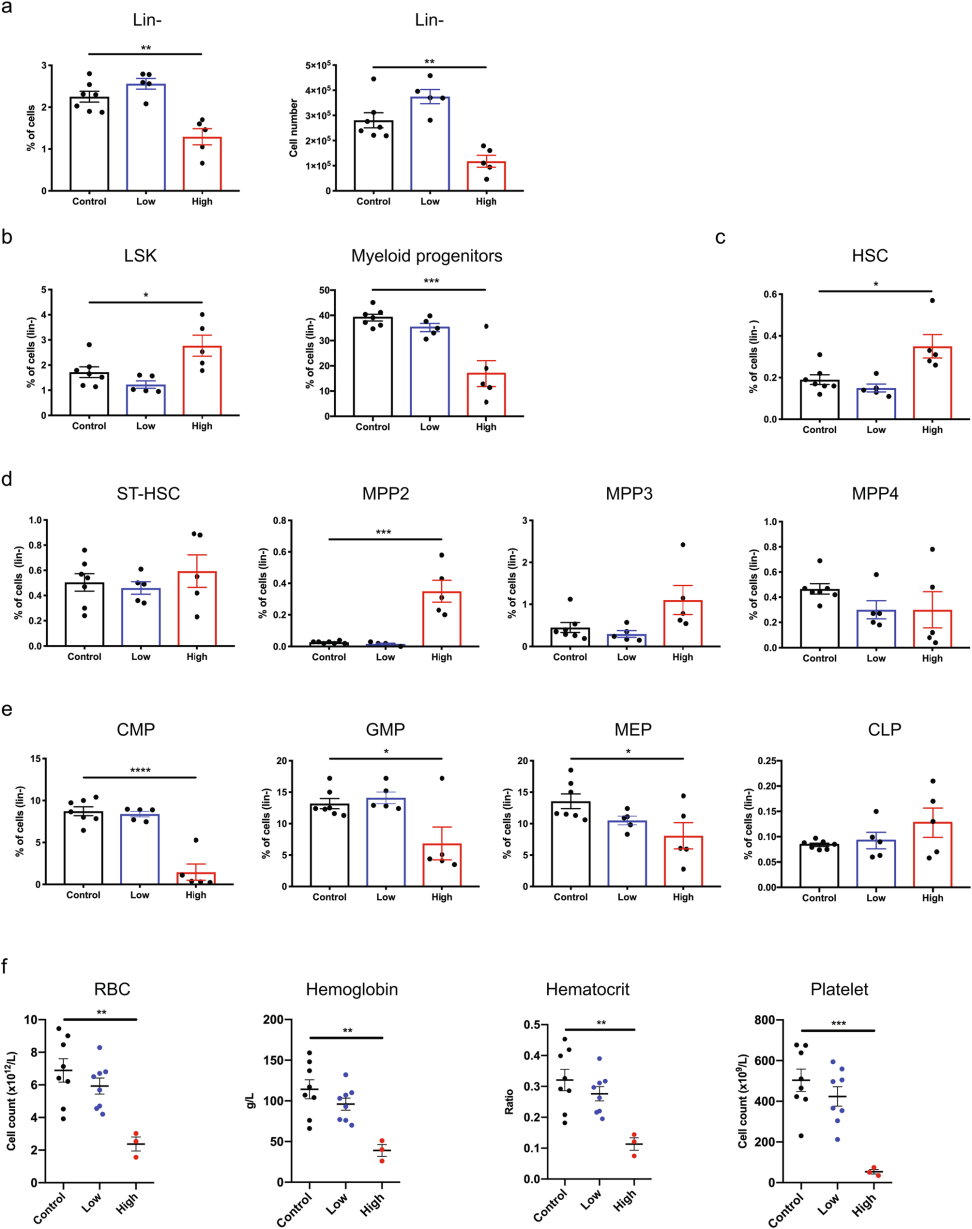
B-ALL alters the HSPC compartment in the bone marrow of leukemia-bearing mice. (**a**) Percentage (left) and number (right) of lineage negative (lin^−^) cells. (**b**) Percentage of LSK cells and myeloid progenitors. (**c**) Percentage of HSCs. (**d**) Percentage of multipotent progenitors. (**e**) Percentage of CMPs, GMPs, MEPs and CLPs. (**f**) Red blood cell count, hemoglobin, hematocrit and platelet count in the peripheral blood of leukemia-bearing mice (n = 3–8). (**a**–**e**) *n* = 5–7. Throughout, *p < 0.05, **p < 0.01, ***p < 0.001, ****p < 0.0001. Error bars represent mean ± SEM.

Our single-cell data suggests that leukemia progression changes the molecular profiles of the hematopoietic cell populations in the bone marrow. While we have previously shown that B-ALL impairs hematopoiesis in bone marrow^9^, the changes in the HSPC compartment have not been explored. Thus, we enumerated subpopulations of HSPC in mice with low (< 1% at day 6) and high (> 40% at day 18) disease burden in the bone marrow (Supplementary Fig. 18)^27^. We identified that mice with a high leukemia burden had a reduction of the lineage negative (lin^−^) population (Fig. 5a), suggesting that leukemia development significantly impairs the hematopoietic progenitors and stem cells. We observed a higher proportion of the LSK (lin^−^Sca1^+^cKit^+^) population and a lower proportion of myeloid progenitors within the lin^−^ fraction (Fig. 5b). Furthermore, we found that expansion of the LSK compartment in mice with a high leukemia burden resulted in a significantly higher proportion of the HSC and multipotent progenitor (MPP) 2 populations (Fig. 5c,d). The observed increase of the MPP2 population and corresponding drop in myeloid progenitors suggests that leukemia development impairs MPP2 to myeloid progenitor differentiation. In addition, we recorded a significantly lower proportion of the CMP, GMP and MEP populations but not CLPs in mice with a high leukemia burden (Fig. 5e). Finally, we enumerated mature hematopoietic cells in the peripheral blood to show that mice with a high leukemia burden showed reductions in red blood cell count, hemoglobin, hematocrit and platelet counts (Fig. 5f), recapitulating the clinical symptoms of anemia and thrombocytopenia in children diagnosed with B-ALL.

## Discussion

The tumor microenvironment influences disease progression and response to therapy. Understanding the tumor microenvironment can lead to identification of novel therapeutic targets. Previous studies have shown that development of B-ALL alters immune cell constitution and the BMM^9,28–30^. In this study, we dissected responses of normal bone marrow immune cells during early stages of B-cell leukemia development at a single-cell level.

We found reduced expression of *Jun*, *Junb*, *Jund*, *Fos* and *Fosb*, all members of the AP-1 transcription factor complex, particularly for pro- and pre-B cells at day 3 of disease progression. AP-1 has been implicated across a wide range of cancers, where it has been reported to be both upregulated or downregulated^31^. This points to opposing roles of AP-1 depending on the cancer type, where increased expression leads to tumor development and reduced expression leads to loss of tumor suppression^32^. In AML, reduced expression of *Junb* and *Jun* was found to be associated with myeloid leukemogenesis through loss of regulation of differentiation, programmed cell death and cellular proliferation^33^. Given the reduced expression we see across these genes, it is likely that AP-1 is similarly acting as a suppressor in our disease model.

For pro- and pre-B cells we also found *Cdkn2a* and *Tmem119* to be over expressed at day 3 of disease progression but not at other time points. In ALL *Cdkn2a* is a tumor suppressor involved in cell cycle regulation^18,19^, and in our disease model there was an acute response to the disease during the early stages of leukemia development, but this response was attenuated as the disease progressed. *Tmem119* is involved in osteoblast differentiation^20^, and we have previously identified that the number of osteoblastic cells decreases during disease progression^9^. Similarly to *Cdkn2a*, it points to an initial response to the disease that is attenuated during disease progression.

Finally, differentially expressed genes for pro- and pre-B cells at day 3 were enriched for regulation by NELFE, MYC and BCL11A. Disease progression in hepatocellular carcinoma was found to be associated with global transcriptomic imbalance due to oncogenic activation of NELFE and enhanced MYC signaling^21^. BCL11A has been implicated in hematopoietic malignancies and AML^22,23^. Enrichment for these regulatory factors was only seen in pro- and pre-B cells at day 3, one of the first signs of induced changes in global transcription in this model.

GMP were found to have reduced expression of *Neat1* at all time points of disease progression other than day 12. Loss of *Neat1* has been shown to induce global transcriptome changes that enable transformation and cancer initiation in oncogene-expressing fibroblasts^34^. In hematological malignancies, *Neat1* has previously been described as a tumor suppressor, with reduced expression associated with multidrug resistance in leukemia and impairment of myeloid differentiation in acute promyelocytic leukemia^25,26^. Our data suggests that *Neat1* also exerts a suppressor function in GMP, although further investigations are required to identify the global effects it may induce.

Intercellular communication networks revealed that monocytes communicate mostly with ST-HSC & MLP and MDP which are monocyte precursor cells. The strong communication between monocytes and MDPs may occur because MDPs give rise to monocytes and the development of leukemia enhances the communication between these cells^35^. Expression of ligands at days 6 and 9 were associated with cell migration and regulation of phosphorylation, whereas expression of receptors were associated with extracellular matrix organization, neutrophil-mediated immunity and cellular response to cytokine stimulus at days 3, 6, 9 and 12. Furthermore, we observed enrichment for GO-BP associated with an inflammatory response in monocytes. Our data supports a recent finding that monocytes are involved in B-ALL development, possibly in response to leukemia-induced inflammation in the bone marrow^30^. For all HSPCs, expression of ligands was associated with enrichment of cytokine-mediated signaling, cell migration and regulation of phosphorylation, though MDP were the only type showing consistent enrichment of these processes at all time points compared to day 0. Enriched processes for receptors expressed by GMP were different at day 12 when compared to all other time points, with decreased activity evident for processes such as apoptosis, cell adhesion and B cell proliferation, but increased involvement was seen for neutrophil-mediated immunity. MDP were the most communicative during disease progression with changes across many GO-BP for both ligands and receptors. Overall, our results support the notion that the leukemia BMM can alter the activities of HSPCs^36^.

Velocity analysis showed changes in GMP from day 3 onwards, where instead of the continuous direction of movement (trajectory) observed at day 0 from neutrophils through to MDP, we observe a disjointed trajectory with GMP grouping with either neutrophils or other HSPCs. This may explain our observations at day 12 in the intercellular communication networks, where we saw an increase in expressed receptors involved in neutrophil-mediated immunity and a decrease in expressed receptors involved in other stem cell like processes such as regulation of apoptotic process and cell–matrix adhesion. This points to GMP remaining responsive to signals from neutrophils during disease progression, but becoming non-responsive to signals from other cell types. This could also be linked to the reduced expression of *Neat1* that we observed in our differential expression analysis.

Consistent with our previous results^9^, we observed a lower proportion of B cells and a higher proportion of neutrophils, the most abundant myeloid cells, during leukemia development. However, key lineage specific transcription factors (PU.1, GATA-1, IKAROS, PAX5) were not differentially expressed in HSPCs, suggesting that the immunocompetent leukemia microenvironment may contribute to deregulation of hematopoiesis. It is also possible that single-cell transcriptomics was not able to detect changes in expression of these transcription factors due to complex dynamics that are better captured with targeted assays^37^. We further explored the HSPC compartment in our in vivo model. While HSPC suppression has been observed in mice with myeloid malignancy^38,39^, our results display skewing of the LSK compartment in B-ALL. Furthermore, we observed a significant decrease of myeloid progenitors during the late stages of leukemia development and a reduction of MEPs, contributing to the clinical features of anemia and thrombocytopenia that occur during leukemogenesis.

In recent years, emerging evidence supports the concept of leukemia-induced BMM remodeling in myeloid malignancies. We are now also beginning to see this concept evolve in lymphoid malignancies. Additional research to translate the immunophenotypic features of the leukemia microenvironment into therapeutic targets remains imperative. Taken together, our results expand the understanding of the complexity of the immune context and the network of molecular interactions within the bone marrow during B-ALL progression.

## Methods

### Mice and single-cell preparation and sequencing

Eight-week old C57BL/6J mice were purchased from the Animal Research Centre, Perth. Animals were housed under pathogen-free conditions and all studies were approved by the Animal Ethics Committee, Telethon Kids Institute, Perth. Using our previously described non-irradiated immunocompetent BCR-ABL1^+^ B-ALL mouse model, marrow plugs were flushed from femurs and tibias^9^. These long bones were crushed and incubated with 337.5 U/mL collagenase (Worthington Biochemical Corp., Lakewood, NJ, USA) and 40 U/mL DNase I (Sigma-Aldrich, Sydney, NSW, Australia) at 37 °C for 60 min in a shaking water bath. The digested marrow cells and the cells from marrow plugs were pooled together. Bone marrow cells were treated with red blood cell lysis buffer. Cell suspensions were counted using a Countess II FL Automated Cell Counter (Thermo Fisher Scientific, Waltham, MA, USA). For single-cell RNA sequencing experiments, five mice were transplanted with 1000 BCR-ABL1^+^ B-ALL cells via tail vein injection. One mouse was euthanized immediately post-transplantation (Day 0, D0). The remaining animals were euthanized 3 (D3), 6 (D6), 9 (D9), and 12 (D12) days post-transplantation. A BD LSRFortessa X-20 (BD Biosciences, Franklin Lakes, NJ, USA) was used to measure the percentage of leukemia cells in the bone marrow (D0 = 0%, D3 < 0.01%, D6 < 0.01%, D9 = 0.017%, D12 = 4.6%). 10 × 10^6^ cells were cryopreserved in 1 mL of freezing solution (90% fetal calf serum + 10% dimethyl sulfoxide) and the frozen samples were sent to BGI Genomics (Shenzhen, Guangdong, China) for single cell sequencing using the 10× Genomics platform (San Francisco, CA, USA). All experiments were performed in accordance with relevant guidelines and regulations.

### Pre-processing, alignment and clustering of single-cell RNA-Seq samples

Raw sequencing data was processed using the 10× Genomics Cell Ranger pipeline (version 2.1.1) and we set the expected number of recovered cells to 6000. BAM files and Cell Ranger processed data are available at the Gene Expression Omnibus repository under accession number GSE148115. We used *DropletUtils*^40,41^ to infer empty droplets for exclusion for each sample. This method compares each barcodes distribution of counts to the distribution of ambient RNA (i.e. droplets with no cell that may contain small amounts of RNA). If the barcode has a significantly different distribution to the ambient RNA (using Benjamini-Hochberg^42^ corrected *p*-values < 0.01) it is deemed to be a cell-containing droplet. We used *scater*^43^ to calculate quality control metrics for each sample and removed cells with a high proportion of reads mapping to the mitochondrial genome (mean absolute deviation > 3). After filtering we obtained 6079 cells for sample D0; 4405 for sample D3; 5137 for sample D6; 6561 for sample D9 and 4378 for D12.

Filtered data was loaded into Seurat version 2.3.4^44^ and we excluded genes detected in less than 3 cells or with counts of zero across all cells. After filtering there were 14,230 genes for sample D0; 14,454 for sample D3; 14,674 for sample D6; 14,780 for sample D9 and 13,237 for sample D12. We normalized each sample using the NormalizeData() function and implemented the global-scaling log-normalization method with a scale factor of 10,000. The data was scaled using the ScaleData() function and we regressed out the effects of the number of detected molecules per cell and the percentage of reads mapping to the mitochondrial genome. We determined the top 1000 highly variable genes for each sample using the FindVariableGenes() function and removed those that were not found in at least two samples. To identify common sources of variation across our samples we used the RunMultiCCA() function to perform canonical correlation analysis on the highly variable genes. We used the MetageneBicorPlot() function to investigate how many canonical correlation vectors were required to explain the structure in our data and chose vectors 1 to 20. The samples were aligned across the canonical correlation vectors using the AlignSubspace() function which applies dynamic time warping. The RunTSNE() function was used to perform t-distributed Stochastic Neighbor Embedding (t-SNE) dimensionality reduction. Clusters were determined using the FindClusters() function which implements a shared nearest neighbor modularity optimization based clustering algorithm^45^ and we set the resolution parameter to 0.6.

### Cell type classification

*SingleR*^46^ was used to annotate cells with cell type. Cell types are inferred by correlating the gene expression profile of each cell to reference transcriptome datasets of pure cell types. We used reference data generated by the Immunological Genome Project (ImmGen)^47^, which profiled 830 samples of mouse immune cells using microarrays. Basophils, endothelial cells, eosinophils and macrophages were excluded from further analysis because very few cells (less than 5 per sample) were annotated with these cell types. Although the bones were subjected to enzymatic digestion, there were not enough CD45^−^ stromal cells detected for further analysis. To check the biological validity of these annotations we used the Seurat FindConservedMarkers() function to find highly expressed marker genes for each cell type cluster. This function performs pairwise differential expression analysis (using the Wilcoxon rank sum test) of each cell type versus all other cell types and combines the resulting *p*-values using meta-analysis methods. For this analysis we required (1) at least five cells per cell type group, (2) at least 75% of the cells in either group to express the gene, (3) a minimum 50% difference in the percentage of cells expressing the genes between the groups and (4) a minimum two-fold increase in expression. We omitted day 12 from the analysis because many cell types are severely depleted at this time point due to disease progression.

### Differential expression analysis

We performed differential expression analysis for each cell type using the Seurat SubsetData() function to subset the data by cell type and input this cell-type-specific expression to the FindMarkers() function where the Wilcoxon rank sum test was used to test for differences in expression at days 3, 6, 9 and 12 compared to day 0. For this analysis we required (1) at least five cells per time point, (2) at least 10% of the cells at either time point to express the genes and (3) an expression fold change of at least 1.5. We used Bonferroni adjusted *p*-values to determine significantly differentially expressed genes and present natural log fold changes (logFC) in the results. For comparisons where there were at least 20 differentially expressed genes, we performed functional gene set enrichment analysis using Enrichr^48,49^ and we used Benjamini–Hochberg corrected *p*-values when determining significance.

### Intercellular communication networks

Networks were constructed using the approach of Skelly et al.^50^ In particular, we used Table S3 from the publication as our set of 2009 mouse ligand-receptor pairs which were adapted from the human set of ligand-receptor pairs^51^. Similarly to Skelly et al., we required 20% of the cells of each type to express the ligand or receptor for inclusion in our analysis. Intercellular communication networks were constructed by counting links between pairs of cells. A link is formed where a ligand is expressed in one cell type and the corresponding receptor is expressed in another cell type. The R igraph package^52^ was used to plot the networks where the edge width corresponds to the number of links. We determined enrichment of GO Biological Processes 2018 (GO-BP) for all of the expressed ligands and receptors for each cell type at each time point using Enrichr, and significance was determined using Benjamini–Hochberg corrected *p*-values. We plotted significance of GO-BP using the aheatmap() function from the R NMF package^53^.

### RNA velocity

We used velocyto^54^ to estimate the transcription rate and future transcriptional state of cells. This method uses the frequency of unspliced introns to estimate the transcription rate (velocity) of cells. Transcripts with unspliced introns are likely to be recently transcribed, as the presence of introns points to the molecule being a pre-mRNA, rather than a fully processed mRNA. RNA velocity is determined using an inferred steady state model of transcription for each gene, where velocity is the difference between the observed ratio of unspliced to spliced Unique Molecular Identifiers (UMIs) and the expected ratio under the model. For each cell, consideration of velocities across all genes allows estimation of the transcriptional rate and the predicted future state of the cell (typically a few hours into the future). The prebuilt mouse genome annotation file (mm10, version 3.0.0) was downloaded from Cell Ranger (https://support.10xgenomics.com/single-cell-gene-expression/software/pipelines/latest/advanced/references). Repetitive elements were masked using the mm10 GTF annotation file available on the UCSC genome browser (downloaded 30th October 2019). Velocyto was run on the Cell Ranger filtered feature-barcode matrices for each time point using the run10x command and specifying the aforementioned genome and repetitive elements annotation files. Output files were read into Seurat using the ReadVelocity() function from the SeuratWrappers package (https://github.com/satijalab/seurat-wrappers). The data was normalized using the SCTransform() function and principal component analysis was performed with the runPCA() function. Clusters of cells were identified by constructing a shared nearest neighbor graph using the FindNeighbors() function based on the first 20 principal components, followed by use of the FindClusters() function which implements a shared nearest neighbor modularity optimization based clustering algorithm^45^. Uniform Manifold Approximation and Projection (UMAP) dimensionality reduction was performed using the RunUMAP() function on the first 20 principal components. Estimates of velocity were calculated using the RunVelocity() function from the SeuratWrappers package, using 25 nearest neighbors for slope calculation smoothing and the top 2% quantiles of expression for the gamma fit. Velocity estimates were visualized on the UMAP dimensionality reduction with the show.velocity.on.embedding.cor() function from the veloctyo.R package (https://github.com/velocyto-team/velocyto.R), using a square root velocity scale with a neighborhood size of 100 and 40 grid points on each axis, with a minimal cell mass of 15 around each grid point.

### Quantitative reverse transcription polymerase chain reaction (qRT-PCR) expression analysis

Total RNA was extracted from neutrophils using a RNeasy Mini kit with RNase-free DNase I (Qiagen, Hilden, Germany). cDNA was synthesized using SuperScript VILO Master Mix (Thermo Fisher Scientific, Waltham, MA, USA). Quantitative PCR was performed on an ABI 7900HT thermocycler using Taqman Gene Expression Assays (Thermo Fisher Scientific, Waltham, MA, USA) for mouse *Atf3* (Mm00476033_m1), *Klf2* (Mm00500486_g1), *H3f3b* (Mm00787223_s1), *Fos* (Mm00487425_m1) and *Gapdh* (Mm99999915_g1). Relative expression was calculated using the ΔΔCT method normalized to *Gapdh* levels for each individual sample (n = 4 mice per time point) measured in duplicate.

### Flow cytometry and cell sorting (FACS)

All FACS studies were performed using single-cell suspensions, and cells were stained using standard protocols. Bone marrow cells were treated with red blood cell lysis buffer. Cell suspensions were counted using a Countess II FL Automated Cell Counter (Thermo Fisher Scientific, Waltham, MA, USA). Flow cytometry was performed on a BD LSRFortessa X-20 and FACS on a BD FACSAria III cell sorter. BD Horizon Fixable Viability Stain 700 (BD Biosciences, Franklin Lakes, NJ, USA) was used for exclusion of dead cells. To enumerate subpopulations of hematopoietic stem and progenitor cells (HSPCs) including hematopoietic stem cells (HSCs), short-term HSCs (ST-HSCs), multipotent progenitor (MPP) 2, MPP3, MPP4, common myeloid progenitors (CMPs), granulocyte–macrophage progenitors (GMPs), megakaryocyte-erythroid progenitors (MEPs) and common lymphoid progenitors (CLPs)^27,55,56^, bone marrow cells were stained with biotinylated rat anti-lineage (B220, CD2, CD3, CD4, CD5, CD8a, CD19, Gr-1, Ter119) antibodies followed by streptavidin-PE as well as anti-mouse CD34-FITC, CD48-BV421, CD150-BV711, CD16/32-PerCP-Cy5.5, IL7Rα-PE-Cy7, Flk2-APC, cKit-APC-H7 and Sca1-BV510 antibodies. The mCherry^+^ leukemia cells were excluded from analysis. For purification of neutrophils, anti-mouse CD11b-BV605 and Ly6G-APC-Cy7 antibodies were used. The gating strategy for subpopulations of HSPCs is shown in Supplementary Fig. 18.

### Peripheral blood cell analysis

Approximately 50 μL of blood was collected from each mouse via cardiac puncture and stored in BD Microtainer blood collection tubes (BD Biosciences, Franklin Lakes, NJ, USA). Blood cell counts were performed using a Mindray BC-5000Vet Auto Hematology Analyzer (Mindray, Shenzhen, Guangdong, China).

## Supplementary information

Supplementary Information.

Supplementary Table S3.

Supplementary Table S4.

Supplementary Table S5.

Supplementary Table S6.
