# Supplementary material for: The bone marrow microenvironment of pre-B acute lymphoblastic leukemia at single-cell resolution
Source: Sci Rep. 2020 Nov 5;10:19173. doi: 10.1038/s41598-020-76157-4 (PMC7645756; doi:10.1038/s41598-020-76157-4)
Supplement: Supplementary file 1 — Supplementary Information. [file 41598_2020_76157_MOESM1_ESM.pdf]

Supplementary Information

The bone marrow microenvironment of pre-B acute lymphoblastic leukemia at single-cell resolution

Anderson, D., Skut, P., Hughes, A.M., Ferrari, E., Tickner, J., Xu, J., Mullin, B.H., Tang, D., Malinge, S., Kees, U.R., Kotecha, R.S., Lassmann, T. & Cheung, L.C.

Supplementary Table S1: Cell counts (percentages) for each inferred cell type group across samples. CLP=common lymphoid progenitor, STSL=short term repopulating cells, MLP=multilineage progenitors, GMP=granulocyte-macrophage progenitors, MDP=monocyte-dendritic precursors, CDP=common dendritic cell precursors, ETP=early T cell precursors.

	**D0**	**D3**	**D6**	**D9**	**D12**
**B cells (CLP)**	31 (0.5%)	43 (1.0%)	28 (0.5%)	61 (0.9%)	16 (0.4%)
**B cells (Pro-B)**	165 (2.7%)	152 (3.5%)	174 (3.4%)	220 (3.4%)	37 (0.8%)
**B cells (Pre-B)**	151 (2.5%)	221 (5.0%)	266 (5.2%)	265 (4.0%)	19 (0.4%)
**B cells**	788 (13.0%)	220 (5.0%)	595 (11.6%)	488 (7.4%)	29 (0.7%)
**Dendritic cells**	83 (1.4%)	144 (3.3%)	176 (3.4%)	169 (2.6%)	6 (0.1%)
**Innate lymphoid cells**	52 (0.9%)	19 (0.4%)	18 (0.4%)	23 (0.4%)	11 (0.3%)
**Macrophages**	15 (0.2%)	18 (0.4%)	39 (0.8%)	41 (0.6%)	1 (0.02%)
**Monocytes**	1,068 (17.6%)	769 (17.5%)	992 (19.3%)	1,049 (16.0%)	619 (14.1%)
**Natural killer cells**	123 (2.0%)	55 (1.2%)	93 (1.8%)	127 (1.9%)	65 (1.5%)
**Neutrophils**	2,800 (46.1%)	2,365 (53.7%)	2,248 (43.8%)	3,449 (52.6%)	3,379 (77.2%)
**Stem cells (STSL)**	4 (0.1%)	12 (0.3%)	5 (0.1%)	20 (0.3%)	1 (0.02%)
**Stem cells (MLP)**	9 (0.1%)	29 (0.7%)	37 (0.7%)	47 (0.7%)	13 (0.3%)
**Stem cells (GMP)**	83 (1.4%)	65 (1.5%)	62 (1.2%)	132 (2.0%)	58 (1.3%)
**Stem cells (MDP)**	51 (0.8%)	38 (0.9%)	25 (0.5%)	60 (0.9%)	30 (0.7%)
**Stem cells (CDP)**	11 (0.2%)	48 (1.1%)	41 (0.8%)	58 (0.9%)	18 (0.4%)
**T cells (ETP)**	14 (0.2%)	7 (0.2%)	1 (0.02%)	5 (0.1%)	3 (0.07%)
**T cells**	565 (9.3%)	137 (3.1%)	254 (4.9%)	277 (4.2%)	56 (1.3%)
**Omitted**	66 (1.1%)	63 (1.4%)	83 (1.6%)	70 (1.1%)	17 (0.4%)
**TOTAL**	**6 079**	**4,405**	**5,137**	**6,561**	**4,378**

Supplementary Table S2: Counts (percentages) of cells selected for downstream analysis by inferred cell type group and sample. ST-HSC & MLP=short term repopulating cells & multilineage progenitors, GMP=granulocyte-macrophage progenitors, MDP=monocyte-dendritic precursors.

	D0	D3	D6	D9	D12
**B cells (Pro & Pre)**	257 (4.2%)	269 (6.1%)	346 (6.7%)	346 (5.3%)	28 (0.6%)
**B cells**	703 (11.6%)	167 (3.8%)	470 (9.1%)	362 (5.5%)	28 (0.6%)
**Dendritic cells**	82 (1.3%)	142 (3.2%)	175 (3.4%)	169 (2.6%)	6 (0.1%)
**Innate lymphoid cells**	52 (0.9%)	19 (0.4%)	18 (0.4%)	23 (0.4%)	11 (0.3%)
**Monocytes**	914 (15.0%)	645 (14.6%)	791 (15.4%)	874 (13.3%)	565 (12.9%)
**Natural killer cells**	84 (1.4%)	32 (0.7%)	62 (1.2%)	88 (1.3%)	56 (1.3%)
**Neutrophils**	2,598 (42.7%)	2,239 (50.8%)	2,081 (40.5%)	3,213 (49.0%)	3,204 (73.2%)
**ST-HSC & MLP**	12 (0.2%)	39 (0.9%)	42 (0.8%)	64 (1.0%)	14 (0.3%)
**GMP**	82 (1.3%)	63 (1.4%)	61 (1.2%)	131 (2.0%)	56 (1.3%)
**MDP**	50 (0.8%)	37 (0.8%)	25 (0.5%)	57 (0.9%)	30 (0.7%)
**T cells**	537 (8.8%)	134 (3.0%)	250 (4.9%)	267 (4.1%)	54 (1.2%)
**Omitted**	708 (11.6%)	619 (14.1%)	816 (15.9%)	967 (14.7%)	326 (7.4%)
**TOTAL**	**6,079**	**4,405**	**5,137**	**6,561**	**4,378**

Supplementary Table S3: Cell-type specific results from differential expression analysis comparing day 3 versus day 0. The Seurat `SubsetData()` function was used to subset the data by cell type and cell-type-specific expression was input to the `FindMarkers()` function to test for differences between the two time points using the Wilcoxon rank sum test. For this analysis we required 1) at least five cells per time point, 2) at least 10% of the cells at either time point to express the genes and 3) an expression fold change of at least 1.5. We used Bonferroni adjusted *p*-values to determine significantly differentially expressed genes and present natural log fold changes (logFC) in the results.

p_val = Unadjusted *p*-value
avg_logFC: =log fold-change of the average expression for day 3 versus day 0
pct.1 = The percentage of cells where the gene is detected at day 3
pct.2 = The percentage of cells where the gene is detected at day 0
p_val_adj = Adjusted *p*-value based on Bonferroni correction

Supplementary Table S4: Cell-type specific results from differential expression analysis comparing day 6 versus day 0. The Seurat `SubsetData()` function was used to subset the data by cell type and cell-type-specific expression was input to the `FindMarkers()` function to test for differences between the two time points using the Wilcoxon rank sum test. For this analysis we required 1) at least five cells per time point, 2) at least 10% of the cells at either time point to express the genes and 3) an expression fold change of at least 1.5. We used Bonferroni adjusted *p*-values to determine significantly differentially expressed genes and present natural log fold changes (logFC) in the results.

p_val = Unadjusted *p*-value
avg_logFC: =log fold-change of the average expression for day 6 versus day 0
pct.1 = The percentage of cells where the gene is detected at day 6
pct.2 = The percentage of cells where the gene is detected at day 0
p_val_adj = Adjusted *p*-value based on Bonferroni correction

Supplementary Table S5: Cell-type specific results from differential expression analysis comparing day 9 versus day 0. The Seurat `SubsetData()` function was used to subset the data by cell type and cell-type-specific expression was input to the `FindMarkers()` function to test for differences between the two time points using the Wilcoxon rank sum test. For this analysis we required 1) at least five cells per time point, 2) at least 10% of the cells at either time point to express the genes and 3) an expression fold change of at least 1.5. We used Bonferroni adjusted *p*-values to determine significantly differentially expressed genes and present natural log fold changes (logFC) in the results.

p_val = Unadjusted *p*-value
avg_logFC: =log fold-change of the average expression for day 9 versus day 0
pct.1 = The percentage of cells where the gene is detected at day 9
pct.2 = The percentage of cells where the gene is detected at day 0
p_val_adj = Adjusted *p*-value based on Bonferroni correction

Supplementary Table S6: Cell-type specific results from differential expression analysis comparing day 12 versus day 0. The Seurat `SubsetData()` function was used to subset the data by cell type and cell-type-specific expression was input to the `FindMarkers()` function to test for differences between the two time points using the Wilcoxon rank sum test. For this analysis we required 1) at least five cells per time point, 2) at least 10% of the cells at either time point to express the genes and 3) an expression

fold change of at least 1.5. We used Bonferroni adjusted *p*-values to determine significantly differentially expressed genes and present natural log fold changes (logFC) in the results.

p_val = Unadjusted *p*-value
avg_logFC: =log fold-change of the average expression for day 12 versus day 0
pct.1 = The percentage of cells where the gene is detected at day 12
pct.2 = The percentage of cells where the gene is detected at day 0
p_val_adj = Adjusted *p*-value based on Bonferroni correction

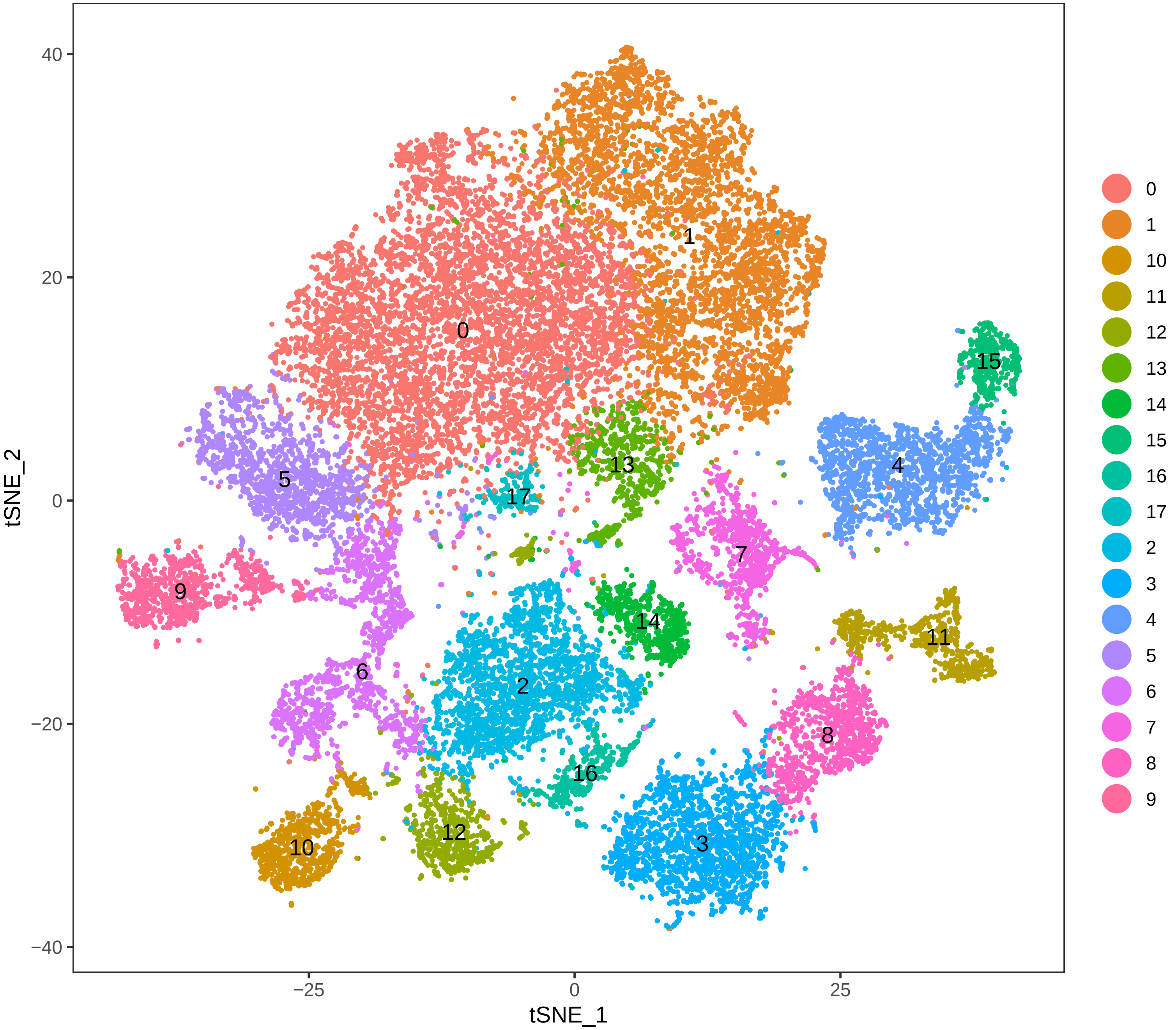

Supplementary Figure S1: Seurat determined clusters for all samples (D0, D3, D6, D9, D12). Cells were clustered by performing t-distributed Stochastic Neighbour Embedding (t-SNE) dimensionality reduction, followed by detection of clusters using the shared nearest neighbour modularity optimisation based clustering algorithm with a resolution parameter of 0.6.

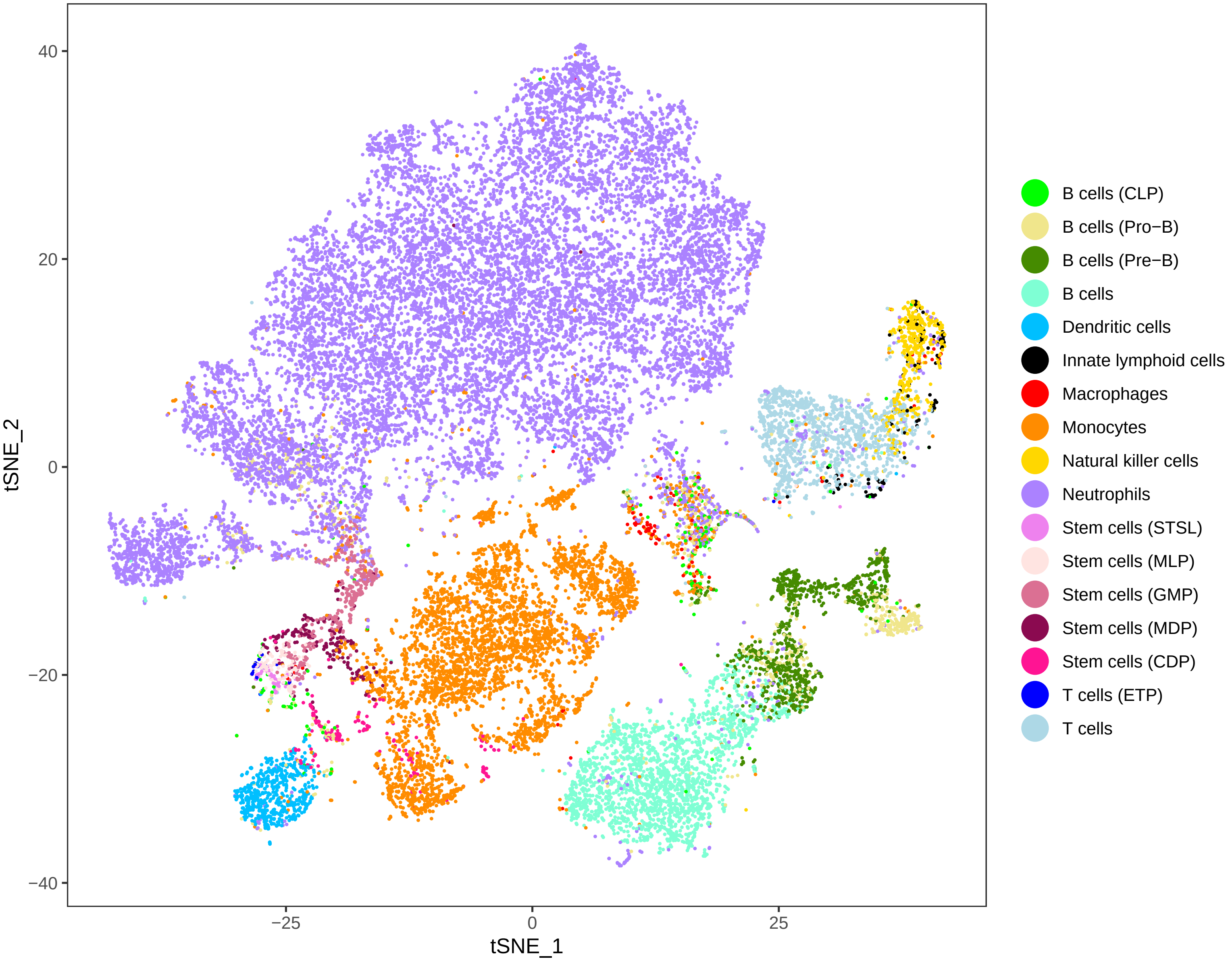

Supplementary Figure S2: Inferred cell type clusters across all samples (D0, D3, D6, D9, D12). Cells were clustered using Seurat and annotated by *SingleR*. 245/26,560 of the cells were omitted because there were too few in the cell type group (i.e. they did not have at least 5 cells at D0, D3, D6, D9 and D12). CLP=common lymphoid progenitors, STSL=short term repopulating cells, MLP=multilineage progenitors, GMP=granulocyte-macrophage progenitors, MDP=monocyte-dendritic precursors, CDP=common dendritic cell precursors, ETP=early T cell precursors.

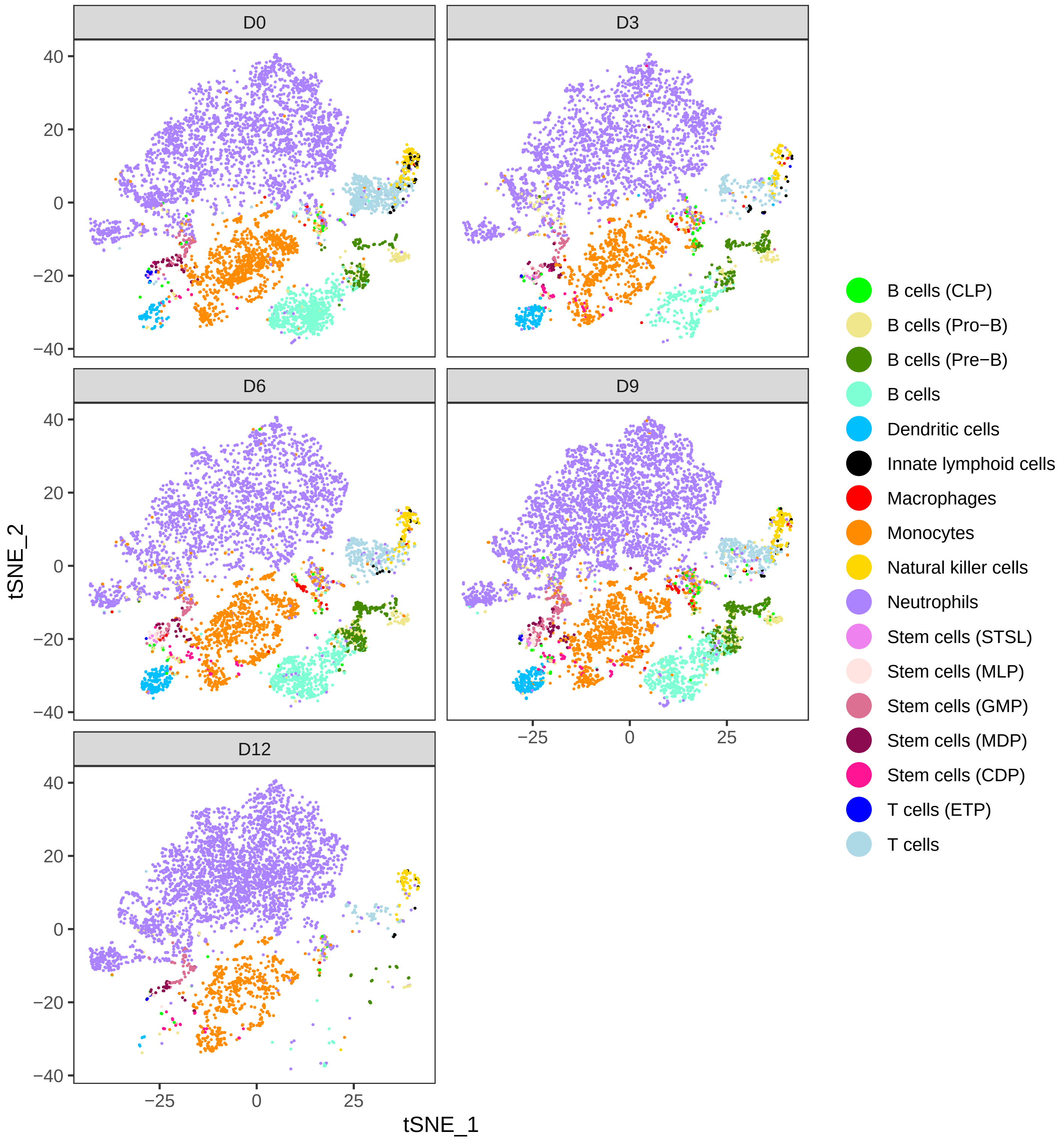

Supplementary Figure S3: Inferred cell type clusters by sample. Cells were clustered using Seurat and annotated by *SingleR*. 245/26,560 of the cells were omitted because there were too few in the cell type group (i.e. they did not have at least 5 cells at D0, D3, D6 and D9). CLP=common lymphoid progenitors, STSL=short term repopulating cells, MLP=multilineage progenitors, GMP=granulocyte-macrophage progenitors, MDP=monocyte-dendritic precursors, CDP=common dendritic cell precursors, ETP=early T cell precursors.

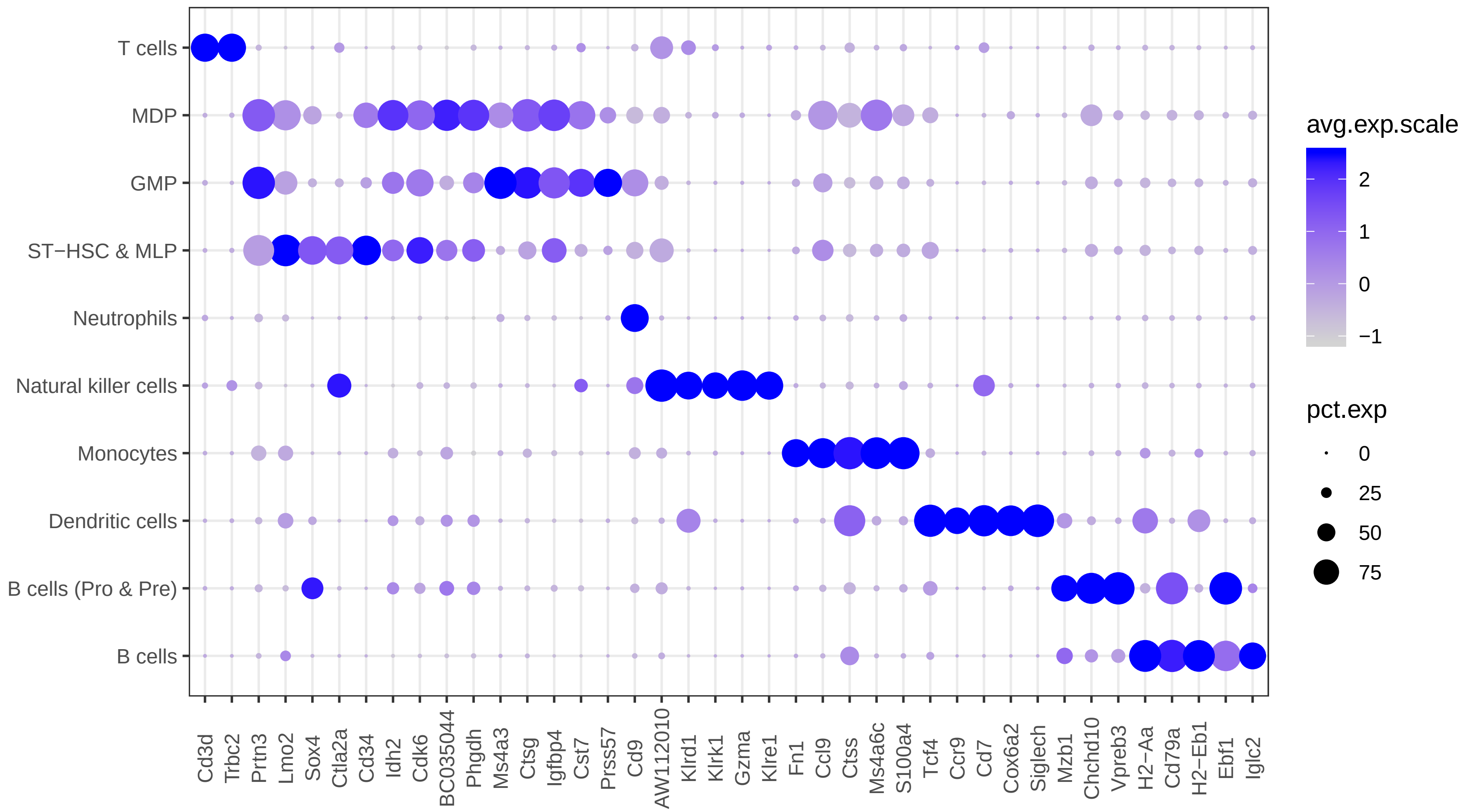

Supplementary Figure S4: Dot plot showing the most (top 5 if available) conserved marker genes for each cell type. The colour of the dot (avg.exp.scale) represents the average expression level of cells within each cell type, where blue is high expression. The size of the dot (pct.exp) represents the percentage of cells expressing the gene within each cell type. We used the Seurat FindConservedMarkers() function to find highly expressed marker genes for each cell type cluster. This function performs pairwise differential expression analysis (using the Wilcoxon rank sum test) of each cell type versus all other cell types and combines the resulting p-values using meta-analysis methods. For this analysis we required 1) at least five cells per cell type group, 2) at least 75% of the cells in either group to express the gene, 3) a minimum 50% difference in the percentage of cells expressing the genes between the groups and 4) a minimum 2 fold increase in expression. We omitted day 12 from the analysis because many cell types are severely depleted at this time point due to disease progression.

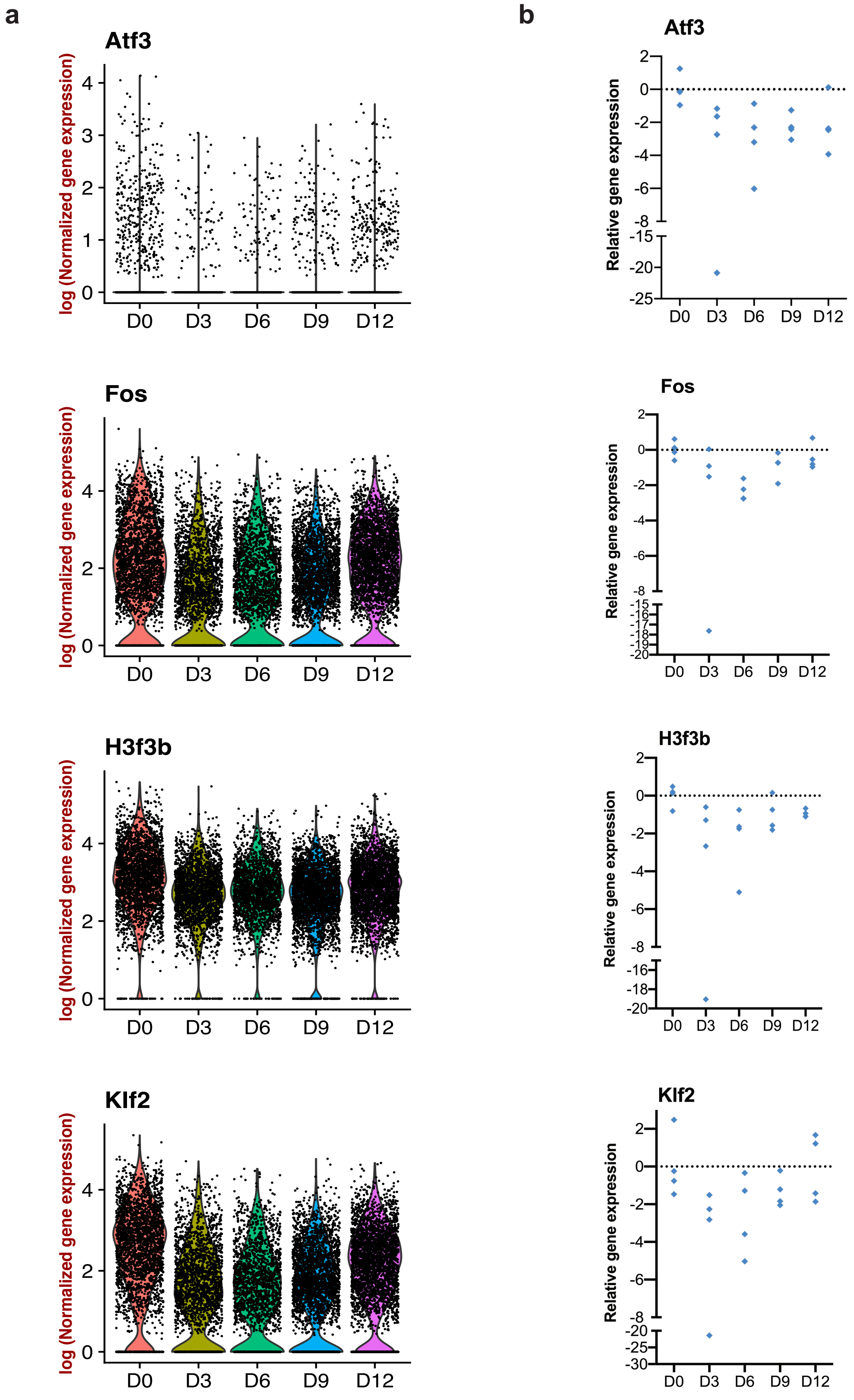

Supplementary Figure S5: Validation of selected genes in neutrophils at different stages of pre-B acute lymphoblastic leukemia development. **(a)** Natural log normalised counts (per 10,000 UMIs) from single-cell RNA sequencing. **(b)** qRT-PCR relative expression calculated using the $\Delta\Delta$CT method normalized to *Gapdh* levels.

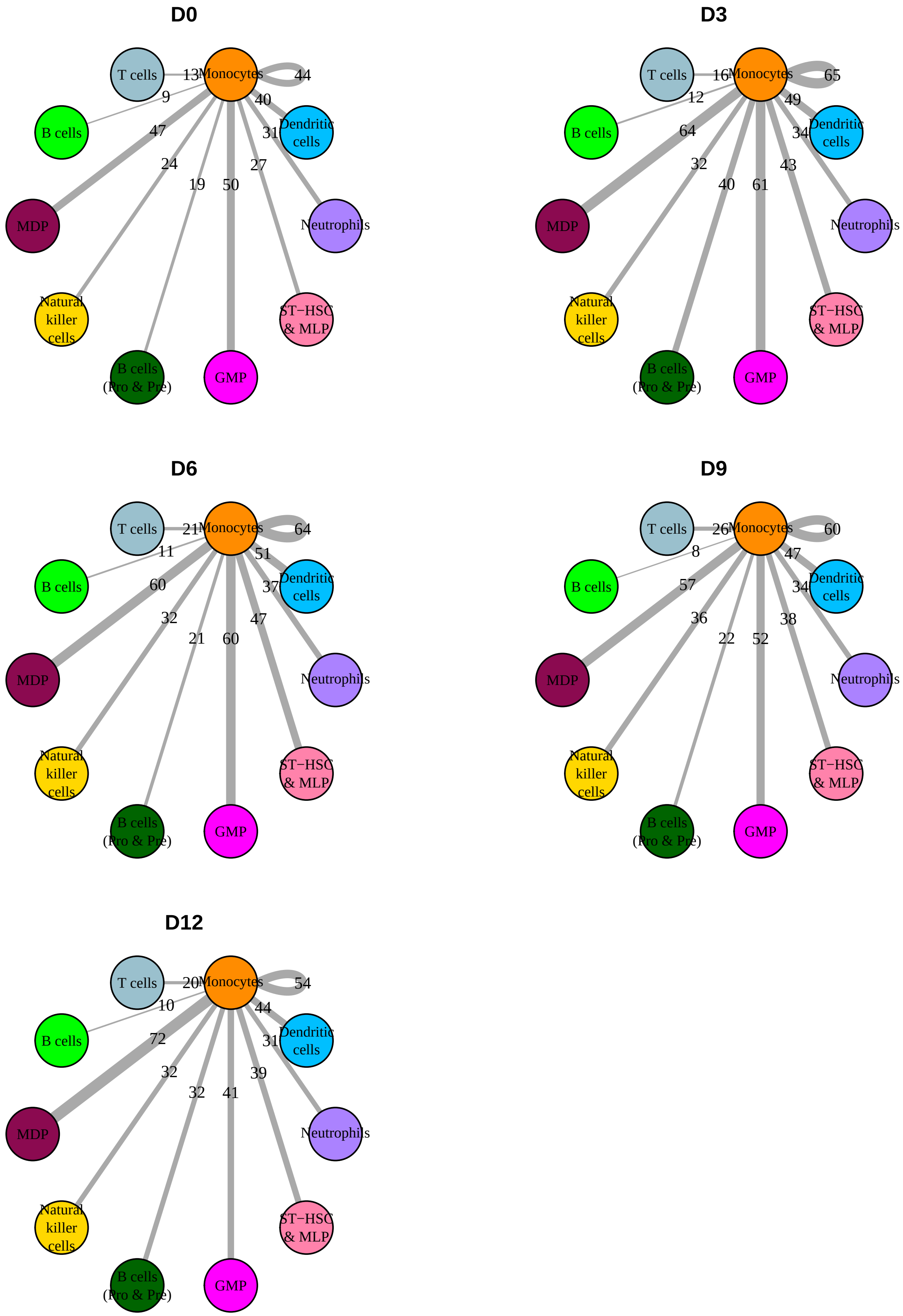

Supplementary Figure S6: Intercellular communication networks for monocytes during disease progression. Edges show the number of ligands expressed by monocytes where the corresponding receptor is also expressed.

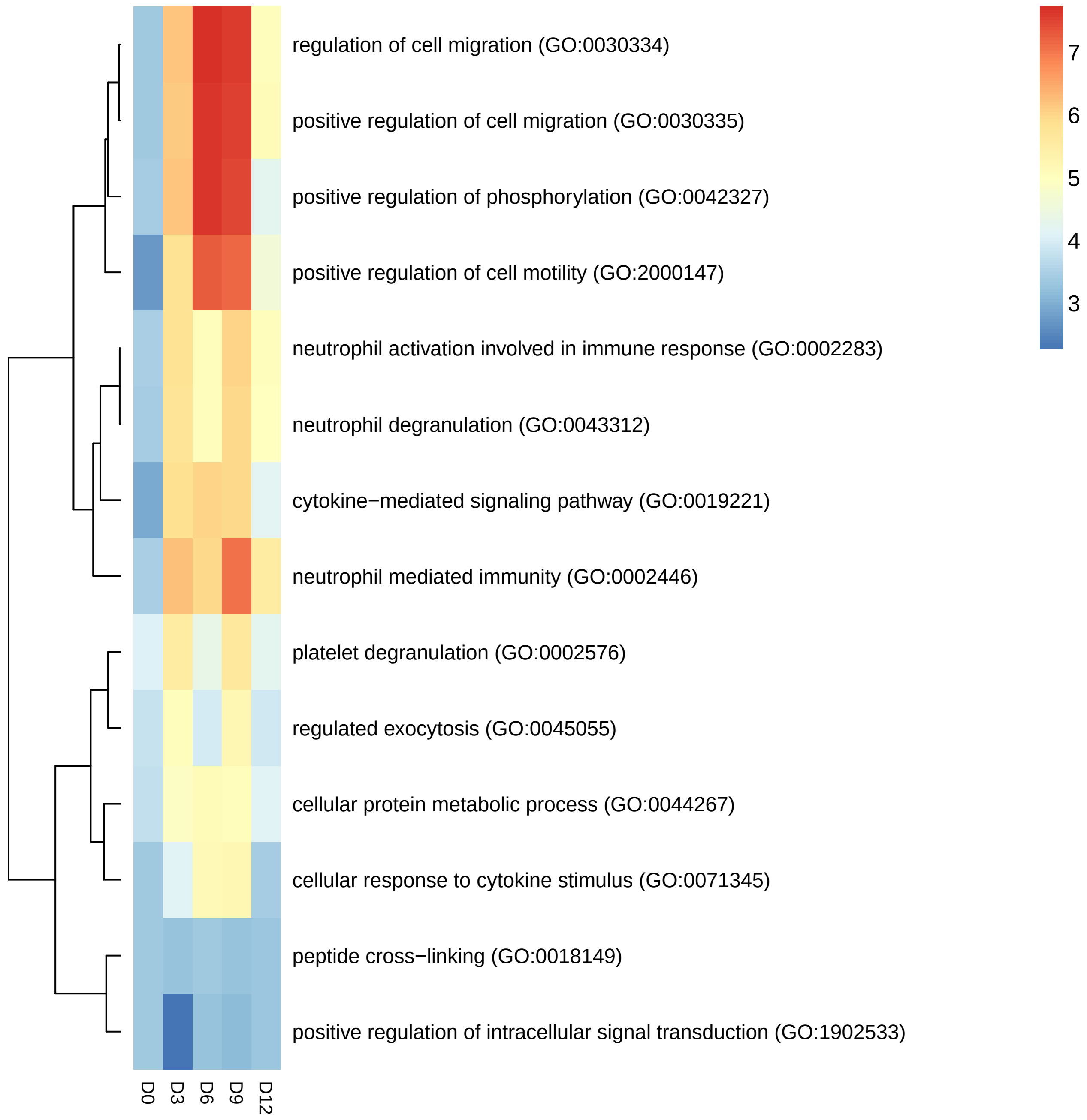

Supplementary Figure S7: Union of top 10 enriched Gene Ontology Biological Processes for ligands expressed by monocytes at each time point. The colour scale represents the -$\log_{10}$ adjusted p-value testing for enrichment of expressed ligands in each gene set.

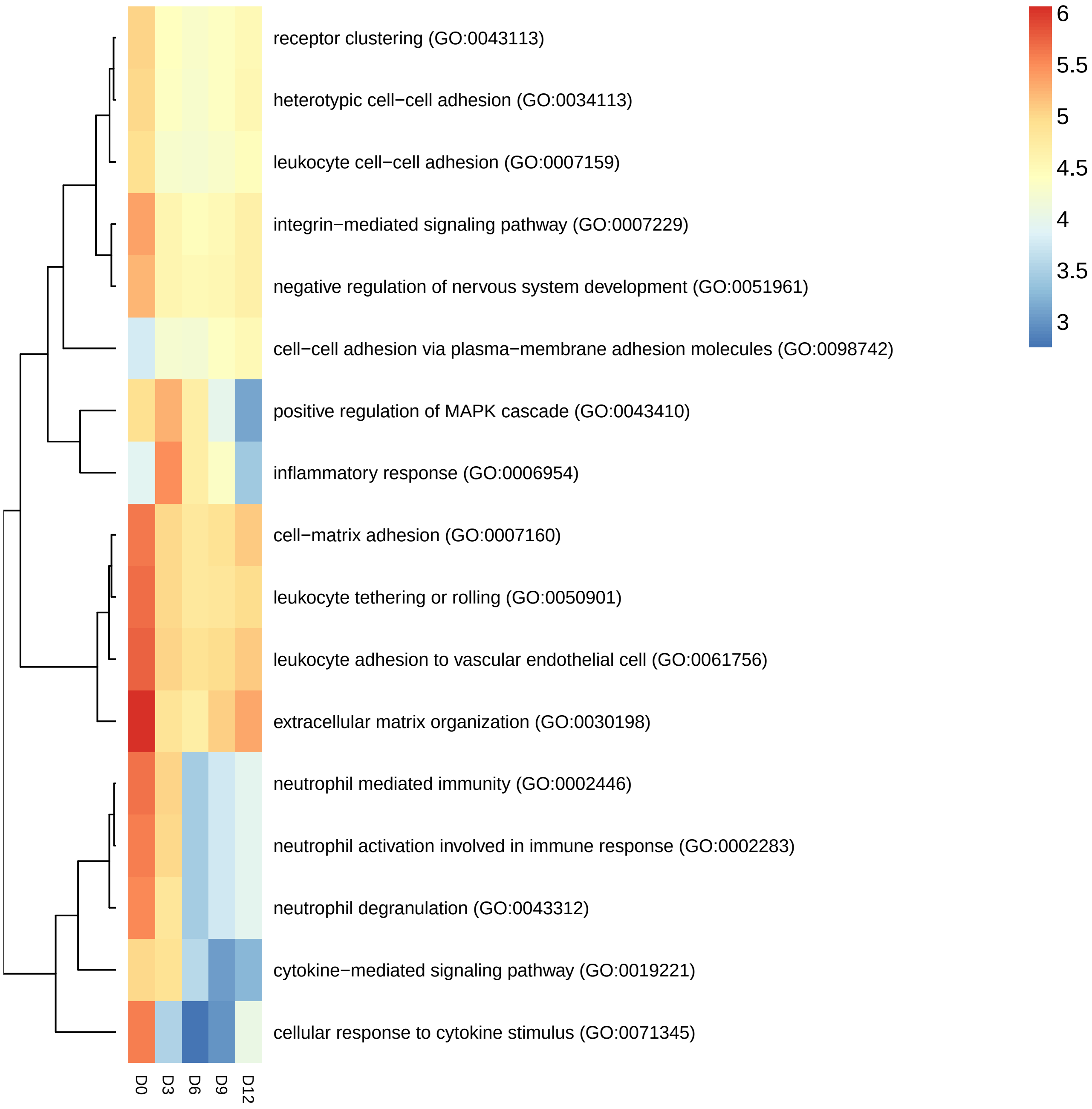

Supplementary Figure S8: Union of top 10 enriched Gene Ontology Biological Processes for receptors expressed by monocytes at each time point. The colour scale represents the -$\log_{10}$ adjusted p-value testing for enrichment of expressed receptors in each gene set.

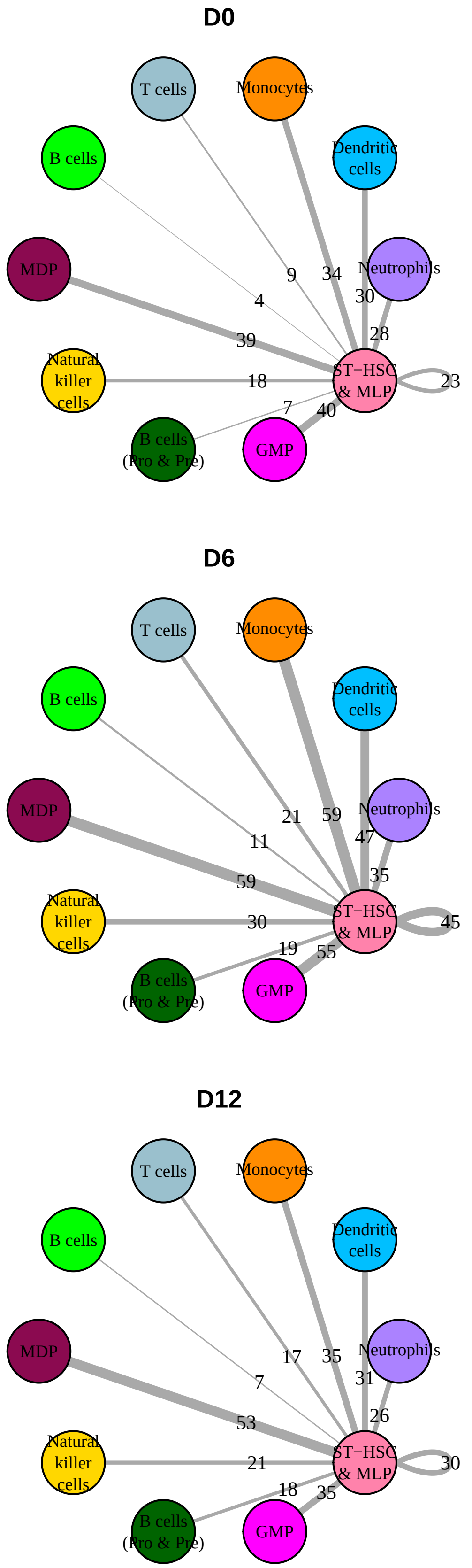

Supplementary Figure S9: Intercellular communication networks for ST-HSC & MLP during disease progression. Edges show the number of ligands expressed by ST-HSC & MLP where the corresponding receptor is also expressed.

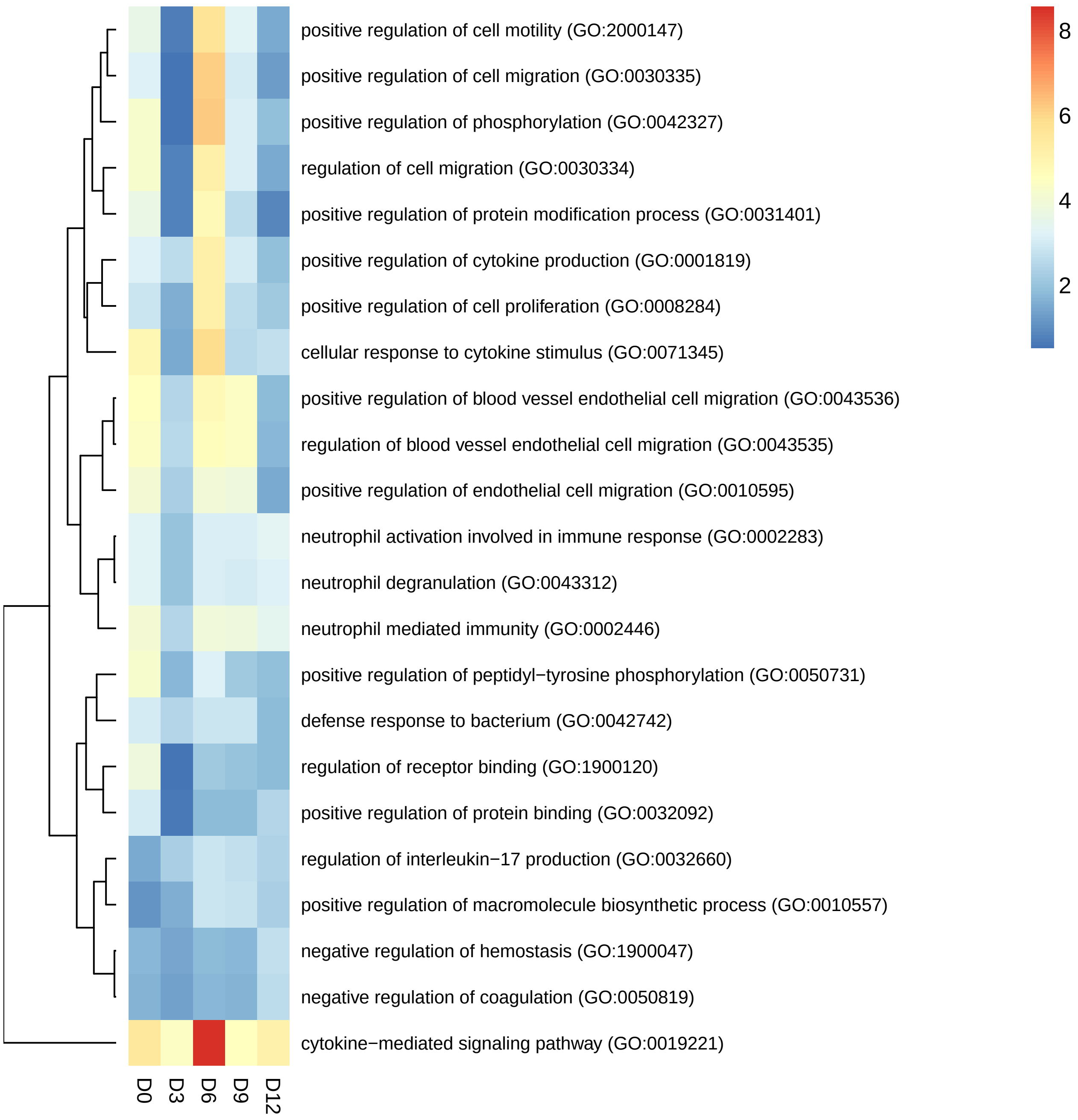

Supplementary Figure S10: Union of top 10 enriched Gene Ontology Biological Processes for ligands expressed by ST-HSC & MLP at each time point. The colour scale represents the -$\log_{10}$ adjusted p-value testing for enrichment of expressed ligands in each gene set.

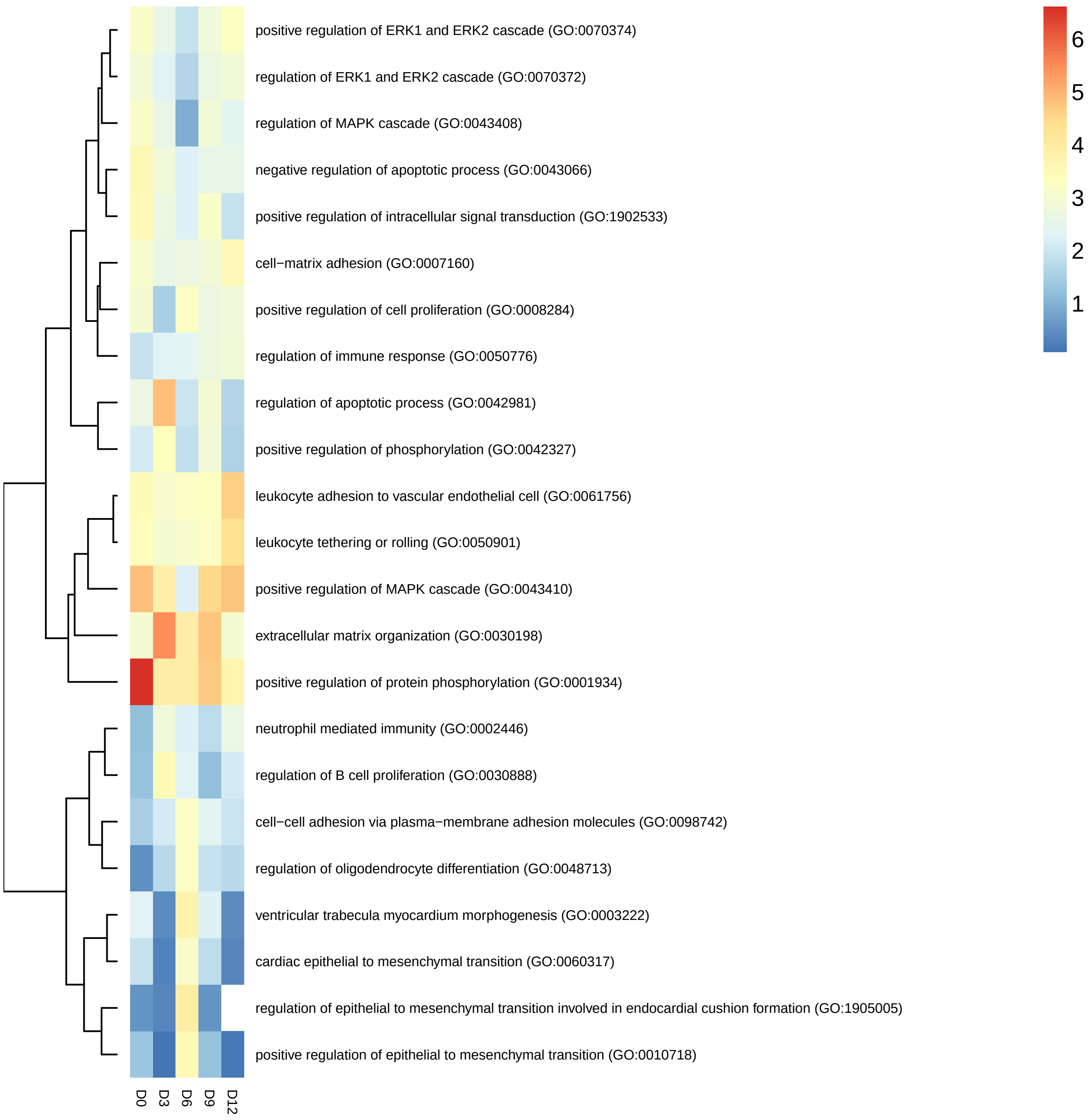

Supplementary Figure S11: Union of top 10 enriched Gene Ontology Biological Processes for receptors expressed by ST-HSC & MLP at each time point. The colour scale represents the -$\log_{10}$ adjusted p-value testing for enrichment of expressed receptors in each gene set.

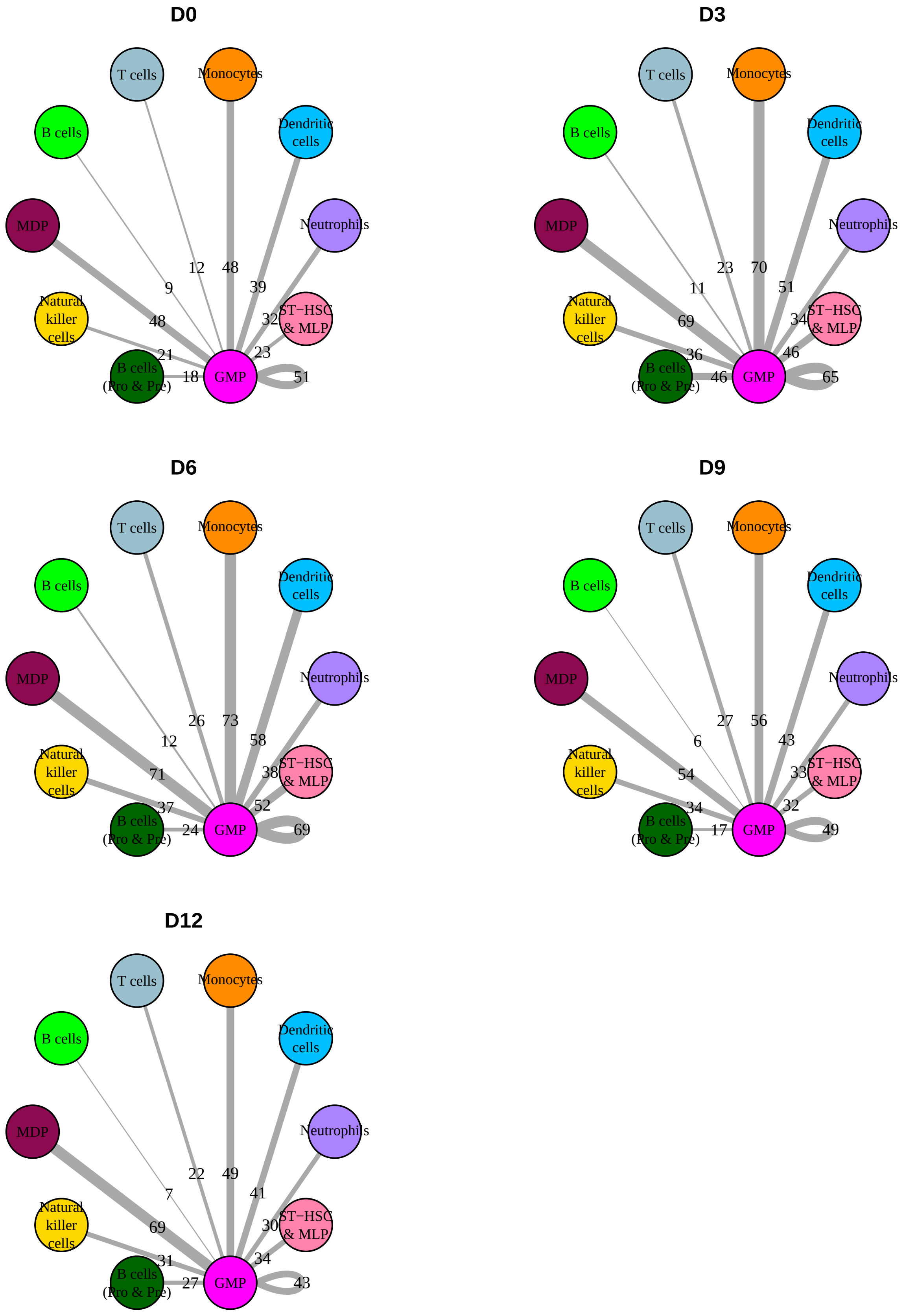

Supplementary Figure S12: Intercellular communication networks for GMP during disease progression. Edges show the number of ligands expressed by GMP where the corresponding receptor is also expressed.

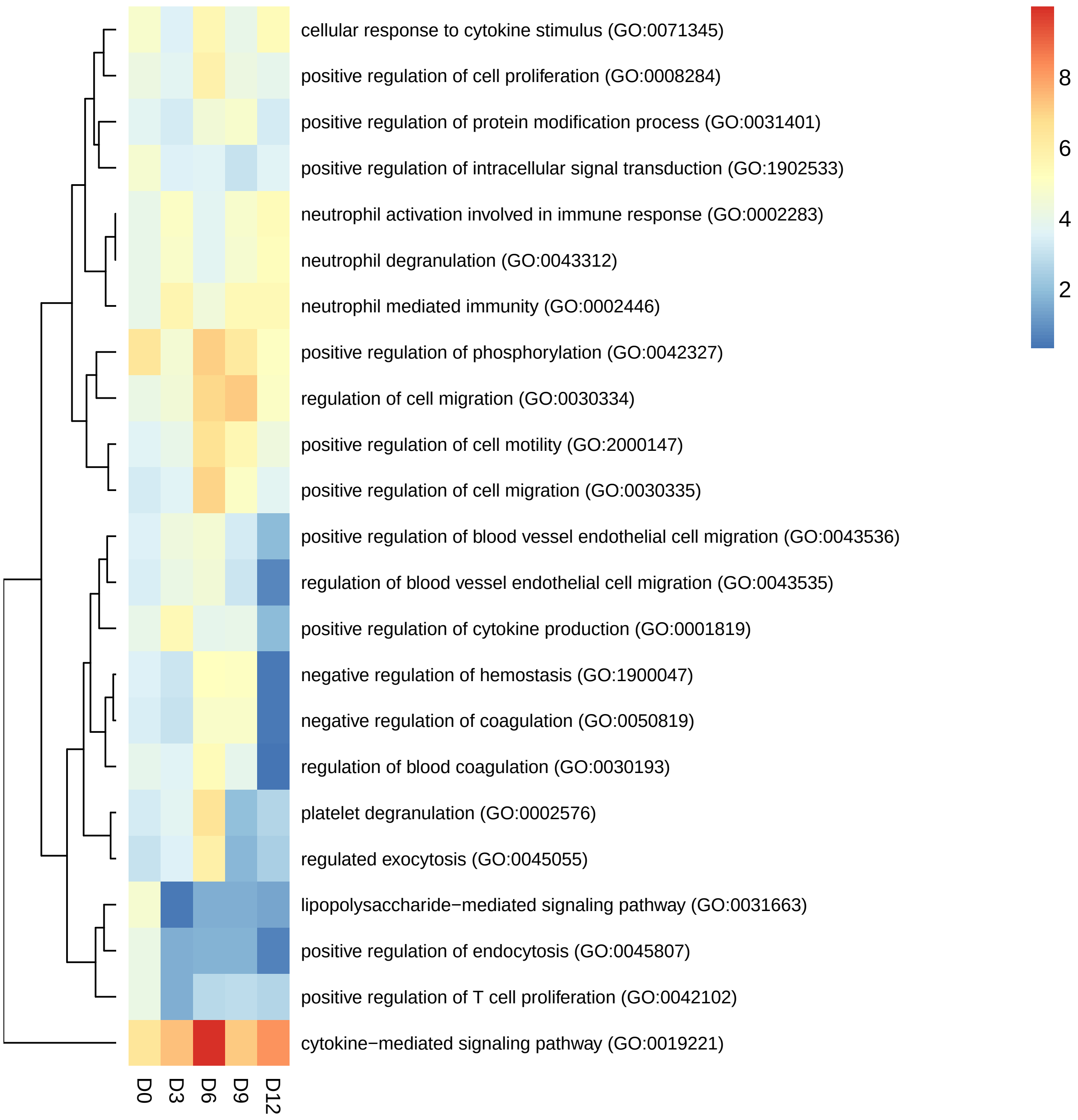

Supplementary Figure S13: Union of top 10 enriched Gene Ontology Biological Processes for ligands expressed by GMP at each time point. The colour scale represents the -$\log_{10}$ adjusted p-value testing for enrichment of expressed ligands in each gene set.

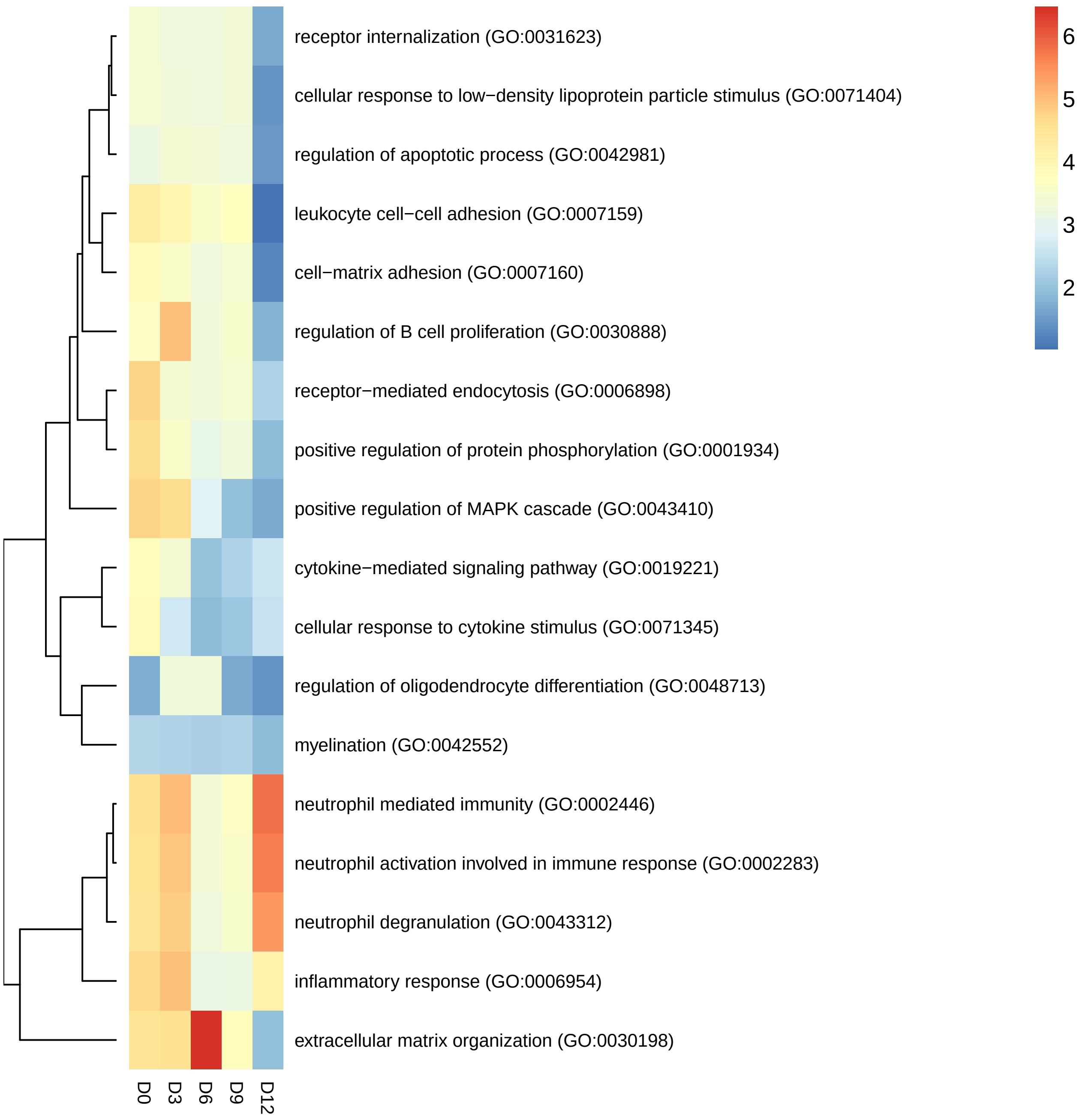

Supplementary Figure S14: Union of top 10 enriched Gene Ontology Biological Processes for receptors expressed by GMP at each time point. The colour scale represents the -$\log_{10}$ adjusted p-value testing for enrichment of expressed receptors in each gene set.

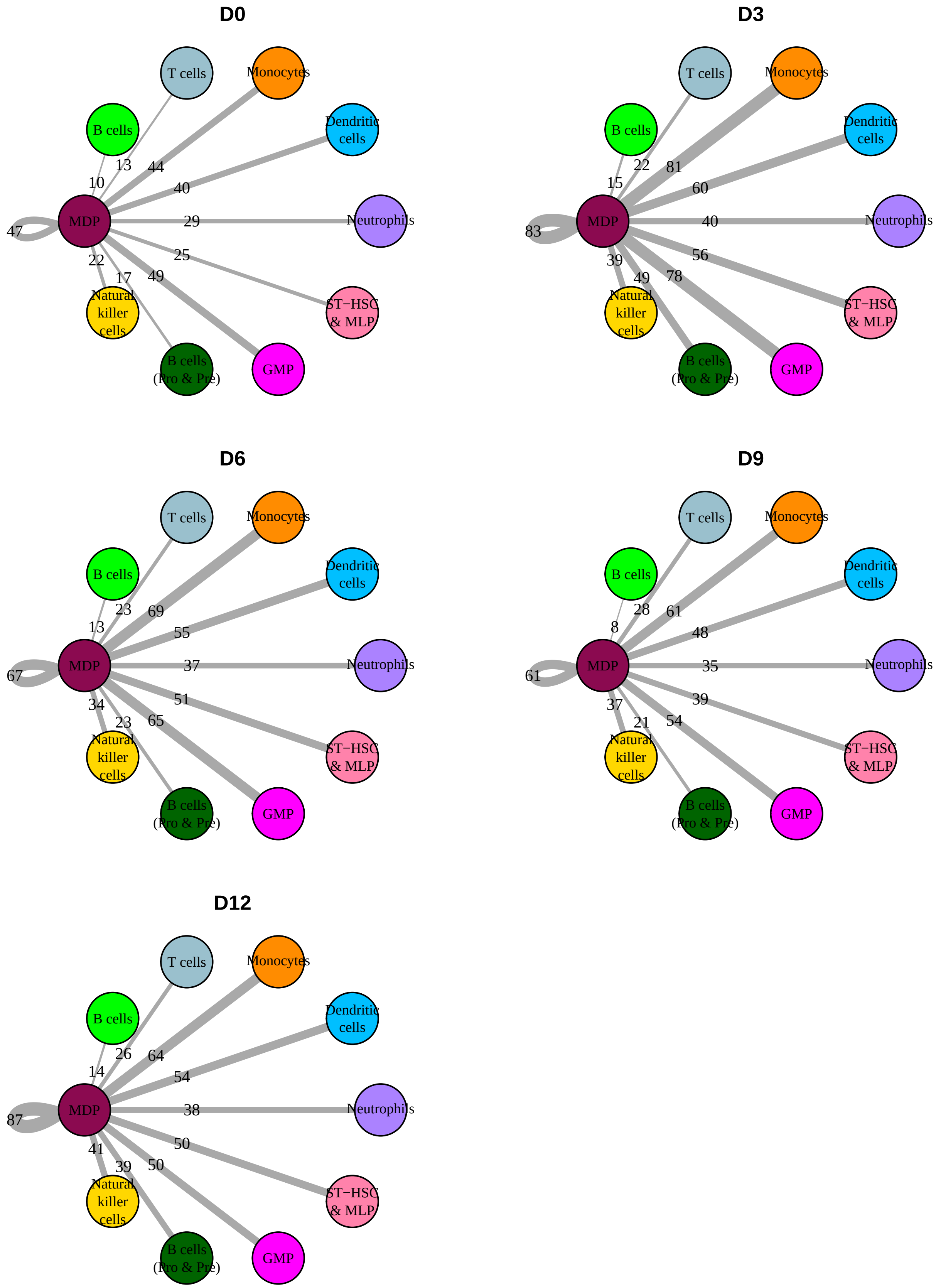

Supplementary Figure S15: Intercellular communication networks for MDP during disease progression. Edges show the number of ligands expressed by MDP where the corresponding receptor is also expressed.

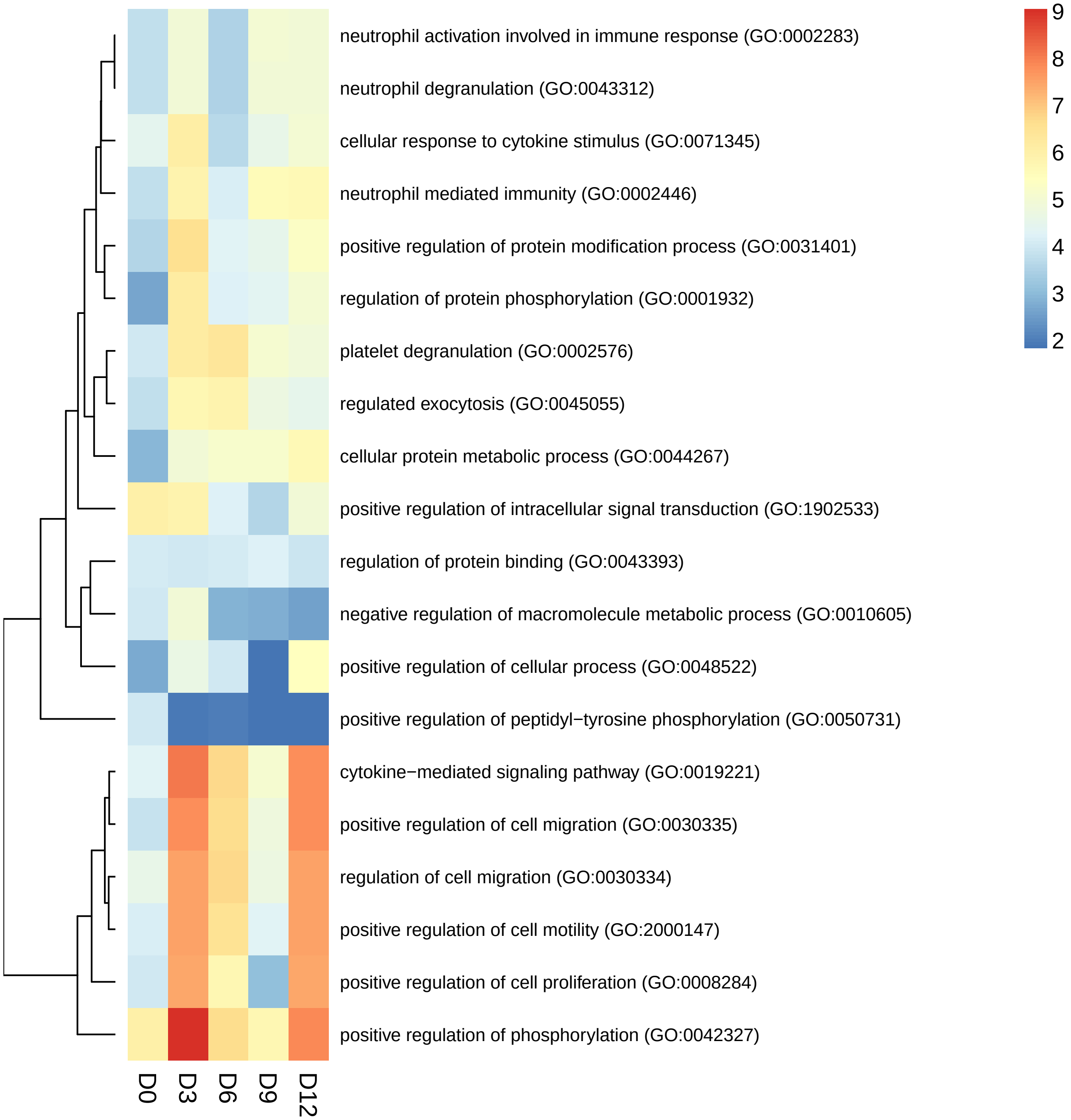

Supplementary Figure S16: Union of top 10 enriched Gene Ontology Biological Processes for ligands expressed by MDP at each time point. The colour scale represents the -$\log_{10}$ adjusted p-value testing for enrichment of expressed ligands in each gene set.

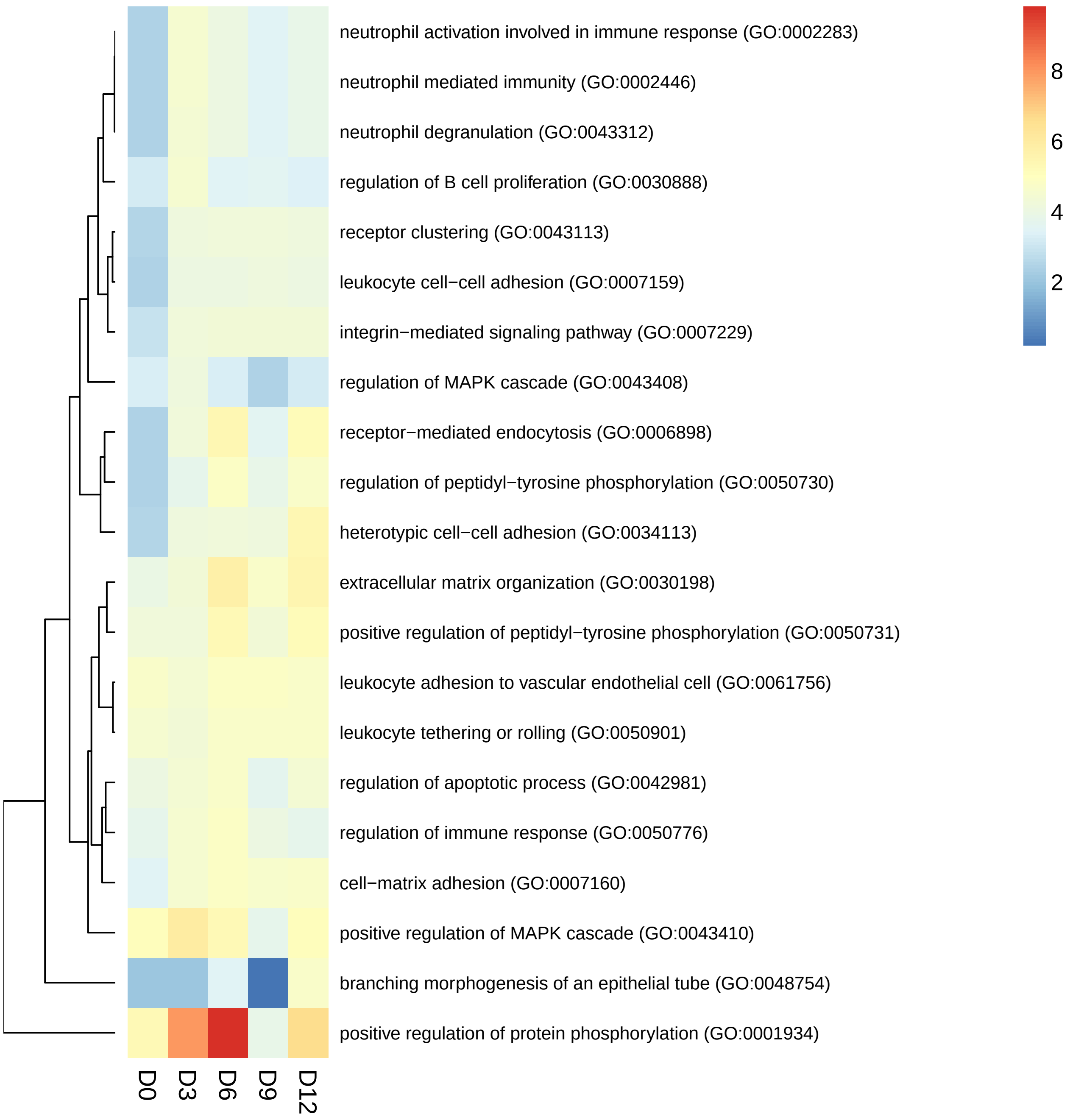

Supplementary Figure S17: Union of top 10 enriched Gene Ontology Biological Processes for receptors expressed by MDP at each time point. The colour scale represents the -$\log_{10}$ adjusted p-value testing for enrichment of expressed receptors in each gene set.

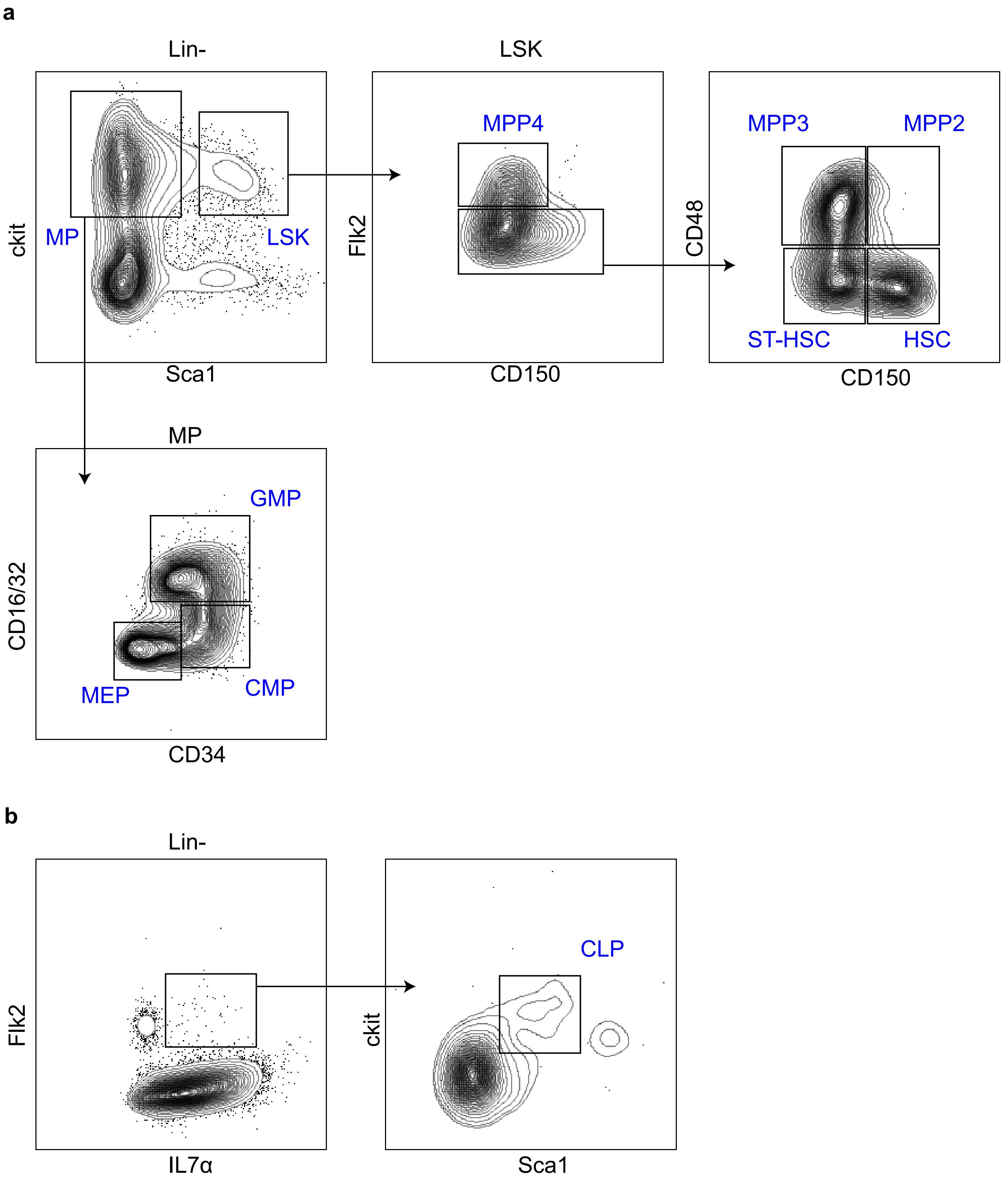

Supplementary Figure S18: Flow cytometric gating strategy for subpopulations of hematopoietic stem and progenitor cells. **(a)** Myeloid progenitors (MP, Lin−ckit+Sca1−), Lin−Sca1+ckit+ (LSK) population, hematopoietic stem cell (HSC, lin−Sca1+ckit+Flk2−CD150+CD48−), short-term HSC (ST-HSC, lin−Sca1+ckit+Flk2−CD150−CD48−), multipotent progenitor cell (MPP) 2 (lin−Sca1+ckit+Flk2−CD150+CD48+), MPP3 (lin−Sca1+ckit+Flk2−CD150−CD48+), MPP4 (lin−Sca1+ckit+Flk2+CD150−), common myeloid progenitor (CMP, Lin−ckit+Sca1−CD16/32(lo)CD34+), granulocyte-macrophage progenitor (GMP, Lin−ckit+Sca1−CD16/32(hi)CD34+), megakaryocyte-erythroid progenitor (MEP, Lin−ckit+Sca1−CD16/32(lo)CD34−). **(b)** Common lymphoid progenitor (CLP, lin−IL7Rα+Flk2+ckit(lo)Sca1(lo)).
